# Quantitative variability of 342 plasma proteins in a human twin population

**DOI:** 10.15252/msb.20145728

**Published:** 2015-02-04

**Authors:** Yansheng Liu, Alfonso Buil, Ben C Collins, Ludovic CJ Gillet, Lorenz C Blum, Lin-Yang Cheng, Olga Vitek, Jeppe Mouritsen, Genevieve Lachance, Tim D Spector, Emmanouil T Dermitzakis, Ruedi Aebersold

**Affiliations:** 1 Department of Biology, Institute of Molecular Systems Biology, ETH ZurichZurich, Switzerland; 2 Department of Genetic Medicine and Development, University of Geneva Medical SchoolGeneva, Switzerland; 3 Department of Statistics and Department of Computer Science, Purdue UniversityWest Lafayette, IN, USA; 4 Department of Twin Research and Genetic Epidemiology, King's College London, St Tomas' Hospital CampusLondon, UK; 5 Faculty of Science, University of ZurichZurich, Switzerland

**Keywords:** heritability, longitudinal variability, plasma biomarkers, SWATH-MS, twin study

## Abstract

The degree and the origins of quantitative variability of most human plasma proteins are largely unknown. Because the twin study design provides a natural opportunity to estimate the relative contribution of heritability and environment to different traits in human population, we applied here the highly accurate and reproducible SWATH mass spectrometry technique to quantify 1,904 peptides defining 342 unique plasma proteins in 232 plasma samples collected longitudinally from pairs of monozygotic and dizygotic twins at intervals of 2–7 years, and proportioned the observed total quantitative variability to its root causes, genes, and environmental and longitudinal factors. The data indicate that different proteins show vastly different patterns of abundance variability among humans and that genetic control and longitudinal variation affect protein levels and biological processes to different degrees. The data further strongly suggest that the plasma concentrations of clinical biomarkers need to be calibrated against genetic and temporal factors. Moreover, we identified 13 *cis-*SNPs significantly influencing the level of specific plasma proteins. These results therefore have immediate implications for the effective design of blood-based biomarker studies.

## Introduction

The effects of genomic variation, modulated by lifestyle and environment, orchestrate the extensive phenotypic variability found in human populations. The quantification of narrow-sense heritability, that is, the proportion of phenotypic variance attributable to additive genetic effects, provides important information for basic and disease biology (Lichtenstein *et al*, 2000; Stranger *et al*, 2007; Emilsson *et al*, 2008; Visscher *et al*, 2008). Within the human population, the narrow-sense heritability of traits can be determined in twin cohort studies. Monozygotic (MZ) twins are genetically identical and thus provide a natural and extremely valuable opportunity to estimate the relative importance of genes and environment by benchmarking MZ phenotype discordances to those of dizygotic (DZ) twins which, on average, share one half of the identical by descent genetic variability (Martin *et al*, 1997).

To date, such studies have been performed at the organismal phenotype, transcript (Grundberg *et al*, 2012; Wright *et al*, 2014), epigenetic (Grundberg *et al*, 2013) and metabolic levels (Nicholson *et al*, 2011; Shin *et al*, 2014), respectively. Proteins predominantly determine the biochemical state of biological specimens and proteomic variation is therefore thought to be closely associated with phenotypic variation, adding a complementary component to the corresponding nucleic acid-based indicators (Aebersold *et al*, 2005; Picotti *et al*, 2013; Wu *et al*, 2013). To date, the reproducible and quantitatively accurate mass spectrometric measurement of proteins across longitudinally collected proteome samples from MZ and DZ pairs of a twin cohort study has been technically challenging due to the high complexity and large dynamic range of human proteome samples, particularly the plasma proteome.

Human blood plasma is the prime source of protein biomarkers and one of the most intensely studied clinical specimens because it can be obtained by minimally invasive methods and contains protein biomarkers that indicate physiological and pathological changes associated with disease (Zhang *et al*, 2007). However, in spite of an enormous number of studies, the clinically important properties of the plasma proteome remain largely unexplored. Specifically, the variability of plasma protein levels in a population, the heritability of protein levels and their longitudinal stability over time remain largely unexplored. Previous relevant population-based proteomic studies have either not been focused on the plasma proteome (Wu *et al*, 2013), or had limited analytical depth (Melzer *et al*, 2008; Kato *et al*, 2011; Lourdusamy *et al*, 2012; Johansson *et al*, 2013) [from 10s to 163 plasma proteins (Johansson *et al*, 2013)] or analytical preference (Enroth *et al*, 2014) and/or limited quantification robustness and outcome reproducibility (Melzer *et al*, 2008; Kato *et al*, 2011).

Here, we used SWATH-MS, an emerging high-throughput targeting mass spectrometry method (Gillet *et al*, 2012; Liu *et al*, 2014; Rost *et al*, 2014), to quantify 342 plasma proteins across 232 plasma samples that were collected with 2- to 7-year intervals from MZ and DZ twin pairs. SWATH-MS essentially combines the analyte throughput of the traditional shotgun or discovery proteomics with the exquisite quantitative accuracy and reproducibility of selected reaction monitoring (SRM), the prototypical quantitative mass spectrometry technique. The data indicate that inherent variability of protein levels varies significantly for different plasma proteins and that the regulation of specific protein levels and biological processes is under tight genetic and/or temporal control. To the best of our knowledge, this is the first study that applies the current, quantitatively accurate mass spectrometric approaches to a twin study for analyzing protein heritability determinants in the (clinically relevant) plasma samples with the unique design of longitudinal sampling.

## Results

### The reproducible quantification of plasma proteins across a longitudinal twin cohort by SWATH-MS

**Figure 1:**
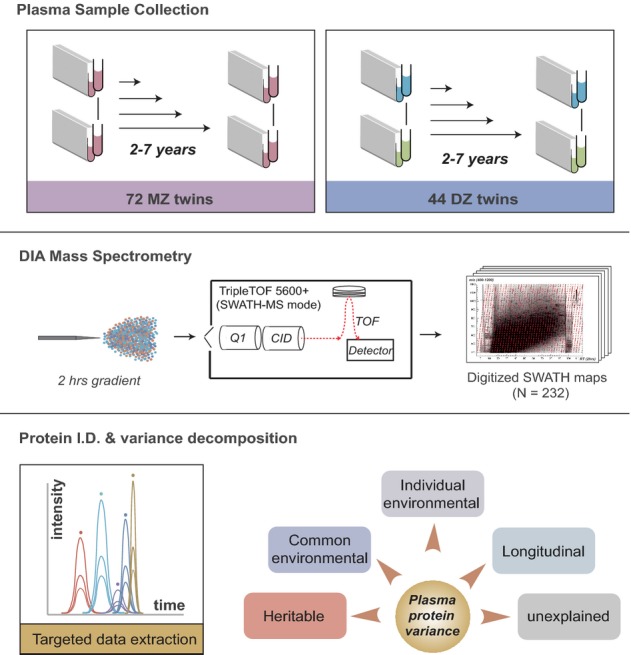
Experimental design The plasma proteomic survey of a longitudinal twin cohort was performed with SWATH-MS, an emerging mass spectrometry technique providing high quantitative accuracy and reproducibility. The observed overall variance of protein abundance was partitioned into four biological contributing factors (heritable, common environmental, individual environmental and longitudinally contributing factors) and unexplained effects using a linear mixed model.

To quantify the levels of human plasma proteins, we applied the newly developed SWATH-MS technique (Gillet *et al*, 2012) in a longitudinal twin cohort. The cohort consisted of 44 DZ and 72 MZ twins from the Twins UK Adult Twin Registry where blood samples were drawn at two different time points (Fig1). Twins were selected at an average age of 57.8 years at the first visit, ranging from 38 to 74 years of age. The time interval between the two samplings was 5.2 ± 1.4 years. The twins had an average age of 63.1 at the time of second visit, ranging from 44 to 78 years of age (see Supplementary Fig S1 for the distribution of actual age in the cohort at the two visits). Fasted plasma samples were collected at identical conditions (see Materials and Methods and Supplementary Table S1). Females were chosen to simplify the experimental design by excluding the gender variance.

**Figure 2:**
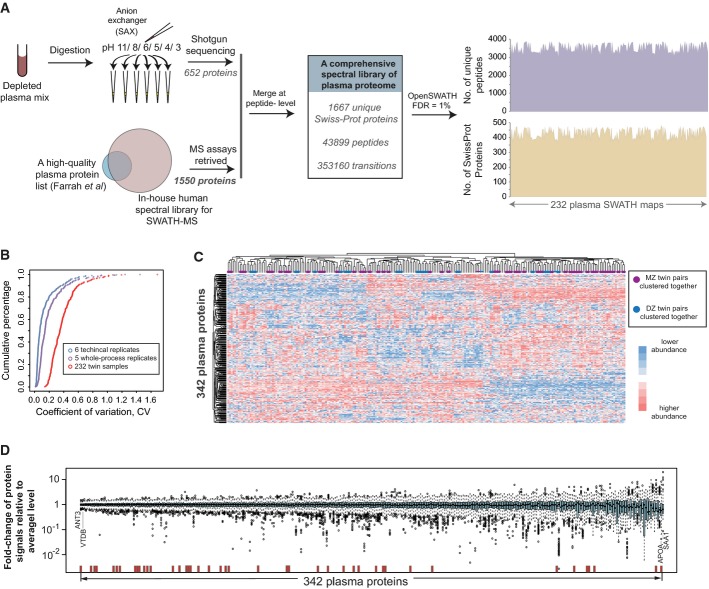
Proteomic identification and reproducible quantification among the twin cohort A The establishment of a comprehensive, specific spectral library of human plasma proteome that is ready for SWATH-MS analysis. The reference spectra were generated *a priori* by extensive shotgun proteomic sequencing of fractionated plasma peptides and complemented with spectra of additional known plasma proteins (Farrah *et al*, 2011).

The data-independent acquisition (DIA) mass spectrometric quantification method of SWATH-MS essentially converts all the peptides ionized from a biological sample into a high-resolution, digital map of fragment ion signals (Gillet *et al*, 2012; Liu *et al*, 2013a) (Fig1). In these maps, specific proteins were monitored via a targeted data analysis strategy, where fragment ion signal groups uniquely identifying a targeted peptide were detected and quantified in each of the 232 SWATH-MS maps. The parameters of the signal group for each peptide, including the fragment ion masses, their relative intensity and chromatographic concordance, the peptide retention time and precursor mass range, constituted a specific assay for each targeted peptide that was prepared *a priori* from a spectral library of the human plasma proteome (Fig2A). Specifically, to generate this spectral library, we deployed comprehensive shotgun proteomic sequencing of the plasma digest of a mixed plasma sample, which was firstly depleted of the 14 most abundant proteins and then fractionated by strong anion exchanger at the peptide level, yielding specific assays for 652 proteins. Further, we included in the library additional MS assays for plasma proteins (Farrah *et al*, 2011) from an in-house SWATH assay compendium for the human proteome (Rosenberger *et al*, 2014). The final combined library contained more than 43,000 peptides, representing 1,667 unique plasma proteins, which represents, as of to date, the largest SWATH-ready spectral library for the human plasma proteome (freely provided with raw data), and therefore maximized the number of identified proteins from the SWATH maps.

B The coefficient of variance (CV) analysis at technical, whole-process experimental and cohort levels.

C Heatmap of hierarchical clustering analysis of the protein vs. sample matrix indicates that plasma proteins were reproducibly quantified by SWATH-MS across the sample cohort.

D The distribution of the fold changes of protein SWATH-MS intensities from their average abundance levels across the cohort is shown as box plots. Antithrombin III (ANT3) and vitamin D-binding protein (VTDB) are shown as examples of the most stable proteins, whereas apolipoprotein(a) (APOA) and serum amyloid A-1 protein (SAA1) are shown as examples of the most variable proteins. The red bars show the positions of the protein analytes whose measurement has been cleared or approved by FDA in human blood.

Using this library and the OpenSWATH software framework (Rost *et al*, 2014), we confidently identified 4,271 unique peptides at an FDR of 1%, corresponding to 534 distinct proteins in all the unfractionated and non-enriched plasma samples (Fig2A and Supplementary Table S2). Their levels in plasma were estimated to cover six orders of magnitude according to human plasma PeptideAtlas database (Farrah *et al*, 2011), reaching, for some proteins, to levels as low as several nanograms per milliliter (Supplementary Fig S2A). On average, 3,520 peptides and 425 proteins were identified from each twin sample. We further filtered these data to select the 1,904 peptides that unambiguously associated with 342 UniProt proteins (Mallick *et al*, 2007) and that were consistently quantified among samples. These peptides constituted a data matrix with only 10.07% missing values across 232 samples and approached 0% missing values after applying the abundance re-quantification algorithm of OpenSWATH (Rost *et al*, 2014) (see Materials and Methods). The dataset contained 42 (that is, about 40% of) protein biomarker analytes whose measurement has been approved by US Food and Drug Administration (FDA) for clinical purpose (hereafter, clinically assayed proteins) assayed in blood (Anderson, 2010). It compares favorably to prior multisample human plasma studies regarding analytical depth (Melzer *et al*, 2008; Kato *et al*, 2011; Lourdusamy *et al*, 2012; Johansson *et al*, 2013), particularly considering that the analytical time was a mere 2.5 h per sample and consumed only 0.015 μl of plasma per SWATH injection, and significantly exceeds the previous studies in terms of reproducibility and quantitative accuracy.

We next sought to assess the properties of the SWATH data. First, we calculated the coefficient of variance (CV) of protein level for each protein. Overall, 84.5 and 76.0% of the proteins quantified in technical and whole-process experimental replicates had CVs of < 25%. The median CV for technical replicates was 7.2% (Fig2B) and for whole-process experimental replicates 14.2%. Second, we compared the quantitative data of SWATH-MS to SRM measurements where we spiked the samples with heavy stable isotope-labeled reference peptides and performed SRM analyses using established SRM assays (Huttenhain *et al*, 2012). As expected, the ratios between light and heavy versions of 41 peptides detected in all the 232 SWATH maps were generally well correlated with SRM results among the samples, with a mean of *R* = 0.85 (Supplementary Fig S2B and C). Third, we performed unsupervised hierarchical clustering analysis (HCA) among SWATH maps (Fig2C). HCA found that 137 (i.e. 59.1% of 232) plasma samples from either the twin pairs or longitudinally sampled individuals (96 samples from 144 MZ twins and 41 from 88 DZ twins) were directly clustered as adjacent nodes, indicating the global proteomic similarity between these samples.

Substantial variability in plasma protein levels was observed among individuals. Overall, 174 (i.e. 50.8% of 342) proteins showed more than tenfold change of SWATH signals between the extremes of the entire cohort. The standard deviation of the fold change of one protein intensity from its average level ranged from 0.1403 for antithrombin III and 0.1465 for vitamin D-binding protein to 1.1936 for apolipoprotein(a) and 1.6871 for serum amyloid A-1 protein, respectively (Fig2D), indicating that protein-level variability is an important feature for different plasma proteins within human population.

### Variance decomposition in the quantitative human plasma protein dataset

**Figure 3:**
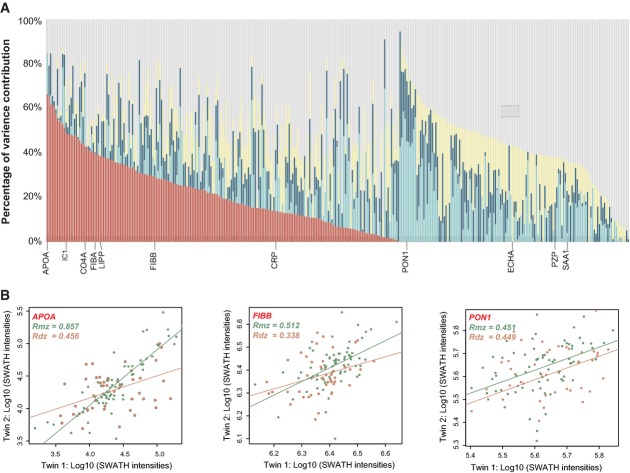
Dissection of the plasma protein-level variability A Histograms of contribution percentage of each biological component (red: heritability; light blue: common environment; dark blue: individual environment; yellow: longitudinal effects; gray: unexplained fraction) determined by a linear mixed model based on the longitudinal twin design. Selected protein names are shown for those clinically assayed proteins with the most heritable levels, for example, APOA, plasma protease C1 inhibitor (IC1), complement C4-A (CO4A), fibrinogen alpha chain (FIBA) and pancreatic triacylglycerol lipase (LIPP), and for the most variable proteins among the cohort, for example, SAA1, APOA, pregnancy zone protein (PZP), trifunctional enzyme subunit alpha (ECHA) and C-reactive protein (CRP).

We took advantage of the longitudinal twin design and utilized a linear mixed model (Nicholson *et al*, 2011) to systematically partition the variance observed for 342 protein levels. The phenotype variance was decomposed into the biological variance (heritable, shared/individual environmental and longitudinal contributing factors) and the unexplained variance (Fig3A). Even though the twins are adult females who normally do not live in the same household, they generally share more habits and lifestyles than non-twin siblings, which are reflected by the term “shared/ common environment”. The unexplained variance generally accounts for 50% of the detected variance in our data and can be associated with variance not reflected by the experimental design (e.g. short-term protein concentration fluctuations, diet effects, etc.) and technical/experimental variations. The mean proportion of heritability, common environment, individual environment and longitudinal process across all the proteins were estimated to be 13.6, 10.8, 11.6 and 13.6%, respectively, of the total phenotypic variance, that is, 25.4, 20.8, 23.0 and 30.8%, respectively, of the biologically stable variance (the total fraction that is explained by the four biological variance origins under our experimental design). The determined heritability (*h*^*2*^) showed good agreement with the protein abundance correlations in MZ and DZ twins. Examples of apolipoprotein(A), fibrinogen beta chain and serum paraoxonase/arylesterase 1 are illustrated in Fig3B. Notably, heritability and common environment are sometimes combined in twin studies as a “family component”, which explains almost half (i.e. 46.2%) of the biological variance in our results. Table1 lists the five proteins most strongly affected by each biological component (see Supplementary Table S3 for variance decomposition results of all proteins). For example, the plasma level of apolipoprotein(A) was determined to be the most strongly heritable (*h*^*2*^ = 0.6633), a finding that is consistent with previous reports (Boerwinkle *et al*, 1992; Lopez *et al*, 2008; Cenarro *et al*, 2014).

**Table 1:** Top proteins mostly affected by the four biological variance components

Swiss-Prot I.D.	Protein name	Gene name	Heritability	Common environment	Individual environment	Longitudinal effects	Unexplained	Direct literature support
Proteins mostly affected by heritability								
P08519	Apolipoprotein(a)	LPA	0.6633* (0.3734–0.9533)	0.1845	0.0000	0.0000	0.1521	Yes
P04220	Ig mu heavy chain disease protein	-	0.6542* (0.5297–0.7787)	0.0000	0.1382	0.0000	0.2077	
O43866	CD5 antigen-like	CD5L	0.6180* (0.2952–0.9408)	0.0409	0.1877	0.0000	0.1534	
Q03591	Complement factor H-related protein 1	CFHR1	0.6149* (0.4902–0.7396)	0.0000	0.0403	0.0099	0.3349	Yes
Q02985	Complement factor H-related protein 3	CFHR3	0.6066* (0.4781–0.7352)	0.0000	0.0249	0.0000	0.3685	Yes
Proteins mostly affected by common environment								
P01877	Ig alpha-2 chain C region	IGHA2	0.0194	**0.6688**	0.2222	0.0082	0.0813	
P00748	Coagulation factor XII	F12	0.0000	**0.5862**	0.1755	0.0764	0.1619	Yes
P31939	Bifunctional purine biosynthesis protein PURH	ATIC	0.0000	**0.5605**	0.1253	0.0065	0.3078	
P01860	Ig gamma-3 chain C region	IGHG3	0.0000	**0.5531**	0.2229	0.0364	0.1876	
O75636	Ficolin-3	FCN3	0.0000	**0.5059**	0.1081	0.0916	0.2944	Yes
Proteins mostly affected by individual environment								
Q99459	Cell division cycle 5-like protein	CDC5L	0.0682	0.0000	**0.5295**	0.1457	0.2566	
P01861	Ig gamma-4 chain C region	-	0.0000	0.4276	**0.5185**	0.0045	0.0495	
P01777	Ig heavy chain V-III region TEI	-	0.1278	0.0155	**0.3881**	0.0794	0.3892	
P02749	Beta-2-glycoprotein 1	APOH	0.0000	0.1009	**0.3807**	0.1324	0.3861	
P43490	Nicotinamide phosphoribosyltransferase	NAMPT	0.0010	0.0000	**0.3594**	0.2425	0.3970	
Proteins mostly affected by longitudinal effects								
Q562R1	Beta-actin-like protein 2	ACTBL2	0.0000	0.0000	0.0000	**0.5354**	0.4646	
P48681	Nestin	NES	0.0000	0.0000	0.0938	**0.4692**	0.4371	
Q16531	DNA damage-binding protein 1	DDB1	0.0000	0.0000	0.1139	**0.4560**	0.4301	
P19367	Hexokinase-1	HK1	0.1918	0.0000	0.0000	**0.4510**	0.3572	
P60174	Triosephosphate isomerase	TPI1	0.0000	0.0000	0.1137	**0.4413**	0.4450	

* Denotes 95% CIs for the heritability estimates (see Supplementary Table S3 for all the proteins). The bold values highlight the significant contribution to the quantitative variance from respective root cause.

B Examples of protein abundance correlations between MZ and DZ pairs. Fibrinogen beta chain (FIBB), Serum paraoxonase/arylesterase 1 (PON1).

We checked if possible modifications of peptides of the identified proteins could affect the variance decomposition results. We found that only < 5% of the peptides included in our model as protein quantitative evidence could be possibly modified according to the Swiss-Prot database with any type modification site reported in the human proteome (Supplementary Table S2 and Supplementary Fig S3A). Even if only these possibly modified peptides were included in the analysis, identical results to those mapped proteins were achieved with respect to determining the contribution of family components to the total variance (*R* = 0.83, Supplementary Fig S3B). This analysis thus suggests that the peptides included in our model for protein quantification are dominated by their naked forms and the peptide-level modification status has an undetectable and negligible effect on our results.

We then compared our results to the only one previous twin study of the similar design, in which 58 plasma proteins were analyzed by using also a female twin cohort from Twins UK but an antibody bead-based technology (Kato *et al*, 2011). The SWATH-MS yielded heritability values for an additional 284 proteins, extending the scope of the study by a factor of six. Most importantly, compared to the antibody technique applied, SWATH-MS achieved a higher degree of reproducibility, obviating the need to remove any outlying samples (Kato *et al*, 2011) (Supplementary Fig S4A–D) and translating into a significantly higher fraction of total phenotype variance that can be explained by biologically stable factors (*P *=* *5.19e-7, Wilcoxon rank-sum test, Supplementary Fig S4E–G).

Intriguingly, in our result the longitudinal factor could explain 13.6% of the phenotype variance, compared to 2.9% reported by Kato *et al* (2011) where the same conceptual variance model as that of our study was used. This discrepancy may be mainly ascribed to the much shorter temporal intervals of sampling used in their study (around 3 months), indicating that the natural aging process together with other longitudinally unstable factors during the ∽5-year period tested in the present study in total uncovered a profound impact of a relatively long-term, temporal changes on human plasma proteomic dynamics. We also carefully checked the existence of other longitudinal factors besides an aging effect (Supplementary Table S1). We found two individuals (i.e. 1.7% of 116 twins) in the cohort who developed cancer between the two visits, and at least 6.9–17.2% of the samples had changed menopausal status at the time of the two visits. A total of 15 (i.e. 12.9% of 116) twins had confirmed type II diabetes before visit one. No individual developed new diabetes type II at visit 2 in this cohort. According to the usage of four types of common medications (corticosteroids, thyroxine, statins and antihypertensives), we found that the twins tended to take more medications at the second visit (an average of 0.38 medications per person at visit 1 versus 0.53 medications per person at visit 2, *P *=* *0.0125). This is consistent with the report from Enroth *et al* (2014) who found a Spearman rho equals 0.29 for the correlation between age and number of medications. In summary, the longitudinal nature plus the twin structure of our sample allowed us to give a quantification of the main causes of variation in protein levels in plasma.

### Differential biological processes preferably regulated by heritability and other biological factors

**Figure 4:**
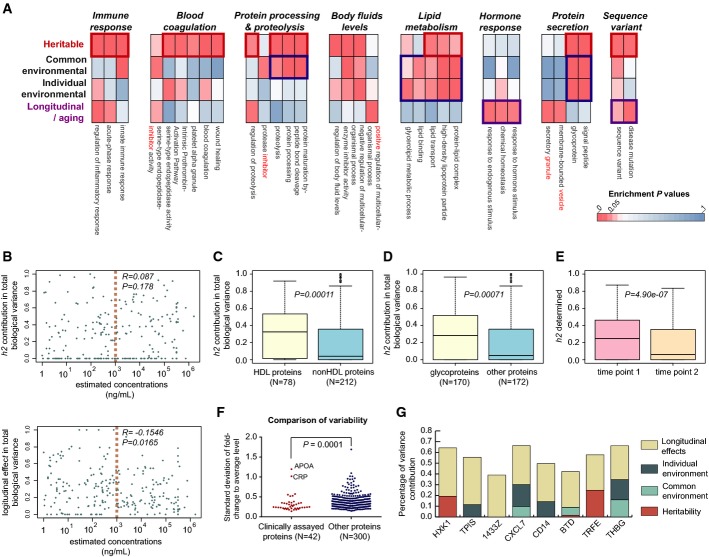
Biological and biomedical insights derived from twin proteomic data A Enrichment analysis of pathways and biological processes regulated by the four biological components was performed. The results were compiled into clusters according to the functional annotation of proteins.

Statistically significant heritability was observed for 80 proteins (i.e. 23% of 342, *h*^*2*^* > *0.25 or *P *<* *0.01). This percentage is close to the result of Johansson *et al* (2013) who measured plasma samples in the parent–children context and thereby determined the abundance levels of 19% of the plasma peptides to be heritable. We confirmed the high heritability of protein level for 21 of the proteins discovered by Johansson *et al* (2013). Additionally, we determined 60 plasma proteins, the level of which was closely associated with longitudinal changes, 52 with familial environment and 47 with individual environment. Among these, 17 proteins appeared to be regulated by both familial and individual environments. To discern the biological processes associated with the four biological sources of variability, we annotated the protein lists by Gene Ontology (GO) and pathway enrichment analysis. This analysis identified several protein functional clusters that are significantly affected by either heritability, environment or the longitudinal effects (Fig4A). For example, a cluster of immune response proteins, consisting of proteins related to the innate immune response and inflammatory regulation (*P*-values between *P* = 0.00032 and *P* = 2.60e-6 for the enrichment significance in all relevant functional processes), the blood coagulation cluster (*P*-values between *P *=* *0.035 and *P* = 0.00019) and a protein-processing cluster (*P*-values between *P *=* *0.040 and *P* = 1.33e-6), were found to be more strongly heritable or familial than associating with individual environment and aging factors. Moreover, the clusters of proteins related to body fluid regulation (*P*-values between *P *=* *0.053 and *P* = 1.16e-5), lipid metabolism (*P*-values between *P *=* *0.065 and *P* = 0.00050) and protein secretion (*P*-values between *P *=* *0.021 and *P* = 1.53e-12) were found to be not only heritable but also heavily interacting with individual environment. Interestingly, the functional cluster of hormone response was under tight regulation of the longitudinal effects (*P*-values between *P *=* *0.030 and *P* = 0.016). These results are consistent with and extend previous literature reports. For example, Souto *et al* (2000) showed that the blood coagulation and fibrinolysis pathways are strongly determined by genetic factors in Spanish families, and Snieder *et al* (1999) noted the importance of genetic dependency of lipid system. Taken together, the twin proteomic data reveal that different biological processes are regulated by genetic control, and environmental or longitudinal factors to different degrees.

B Low correlation between plasma protein levels extracted from PeptideAtlas (www.peptideatlas.org) and their heritability contributing percentages in biological variance indicating the lack of an abundance bias. In contrast to that, concentration variability of more abundant proteins is generally less affected by longitudinal factors. The light red dashed line indicates the protein concentration of 1 μg/ml, which separates the proteins into two abundance classes.

C Comparison of high-density lipoproteins (HDLs) and other proteins, using the heritability contributing percentages in biological variance of the plasma protein levels.

D Comparison of heritability contributing percentages in biological variance between those proteins annotated as glycoproteins and other proteins. *P*-values are determined by Wilcoxon rank-sum test.

E Decreasing trend of heritability control in plasma protein levels along with 5-year longitudinal process.

F Clinically assayed proteins generally show lower quantitative variability compared to other plasma proteins with few exceptions, for example, CRP and APOA.

G Examples of previously reported protein biomarker candidates, the plasma abundance levels of which were highly regulated by longitudinal effects. These include hexokinase-1 (HXK1), triosephosphate isomerase (TPIS), 14-3-3 protein zeta/delta (1433Z), platelet basic protein (CXCL7), monocyte differentiation antigen CD14 (CD14), biotinidase (BTD), serotransferrin (TRFE) and thyroxine-binding globulin (THBG). Data information: *P*-values are determined by Wilcoxon rank-sum test.

### The biological variance dissected for proteins of different plasma concentrations

The systematic dissection of the origins of variance of plasma proteins may provide opportunities for new biological insights. For example, using the estimated concentration levels of plasma proteins from PeptideAtlas (Farrah *et al*, 2011), we investigated if the total variability (represented by standard deviations of the fold change of protein signals relative to the average level) and the relative contributions of the four biological components globally depended on the plasma concentration. The result suggests that most of low abundant proteins are more variable; however, there are a few high abundant proteins whose levels were also highly variable among the cohort, which in turn makes the statistical correlation between variability and protein concentration insignificant (Supplementary Fig S5). Importantly, by analyzing the relative contributions of factors explaining total biological variance, we found that the correlation between plasma protein levels and heritability values was low (*R* = 0.087, *P* = 0.178 for the correlation, Fig4B), suggesting that the concentration levels of more abundant proteins are not necessarily more strongly determined by genes. The variability from both common and individual environmental components has no detectable associations with the protein concentrations. In contrast, concentration variability of more abundant proteins is generally less affected by longitudinal factors such as aging (*P* = 0.0165, Fig4B). This novel finding could thus indicate the limited regulation power of, for example, longitudinal aging processes on highly abundant plasma proteins or that the longitudinal factors preferably control the levels of low abundant proteins.

### The heritability of circulating levels of high-density lipoproteins and other protein classes

We next analyzed the source of variation in a clinically and biologically related set of proteins, those associated with high-density lipoproteins (HDL) (Shah *et al*, 2013). HDL represents a complex, bioactive particle in human plasma that can be isolated by density gradient ultracentrifugation and has been shown to minimally consist of 85 proteins (Shah *et al*, 2013). HDL has a range of roles, such as the promotion of reverse cholesterol transport and modulation of inflammation. Here, we quantified the circulating levels of 92% of putative HDL proteins (that is, 78 out of 85). The data indicate that HDL proteins showed a markedly higher heritability compared to the non-HDL protein quantified in this study (*P *=* *0.00011, Fig4C), suggesting that the biological roles (e.g. cardioprotective effect) of HDL in humans might be under high genetic control (Hegele, 2009).

We repeated the heritability analysis for proteins containing different types of modifications according to human Swiss-Prot database and different domains according to Pfam database. Interestingly, proteins annotated as “Glycoproteins” (FigD, *P* = 0.00071) and as containing “Disulfide bond” were more strongly regulated by genetics and less affected by longitudinal factors compared to other proteins, while the proteins denominated “Phosphoprotein” or containing “Acetylation” showed a reversed regulation trend (Supplementary Fig S6, *P* < 0.01 or *P* < 0.05). We further found that proteins with “V-set domains” seemed to be highly regulated by family component (the combination of heritability and shared environment). These observations might be associated with the protein class functions; for example, proteins with V-set domain in blood are mainly immune proteins that are more strongly heritable (Fig4A). Furthermore, considering the overlapping proteins between annotation classes and the fact that we did not directly measure any protein modification and structure in the current study, we suggest that further direct studies are crucial to conclude whether different protein modifications or structures indeed harbor diverse genetic or longitudinal regulation dependency.

### Genetic contribution to plasma protein-level control between the two longitudinal visits

To study the question whether the genetic contribution to the control of plasma protein concentration levels varied over time, we firstly separated the dataset according to the two longitudinal time points which were on average 5 years apart and then compared the heritability values of all the proteins quantified at the two sampling points. The results indicate (Fig4E) that heritability generally decreased over time during the 5-year process, for the proteins tested (*P *=* *4.90e-07). To corroborate this result, we included all the informative peptides of our proteins (instead of top abundant ones) for the statistical test and obtained the same global trend for majority of the peptides (*P *<* *2.2e-16 based on 1,904 peptides, Supplementary Fig S7A). We then added together the heritability and the common environment factor as family component and observed that the family component still had a decreased trend of contribution to the protein variability between two time points (*P *=* *0.0008, Supplementary Fig S7B). To further investigate this phenomenon, we factored the real age of each individual into the model and observed that the real age had a minimal effect in determining the heritability (correlation of 99%) and in explaining the variance (only explained 0–1% of the variance for most proteins) (Supplementary Fig S7C–H). It is worth mentioning that the real age could consistently explain > 4% of the total variance for only two proteins, fibrinogen beta chain (FIBB) and lumican (LUM), at both visits. Altogether, these results suggest that in these relatively old individuals tested (average age of 57.8 at visit 1), the genetic regulation of many (but not all) plasma proteins decreased along with longitudinal processes or that the environmental factors and other factors unexplained by the linear mixed model have an increased contribution in proteomic variability during the 5-year temporal process.

### Insight for biomarker discovery studies derived by protein variability analysis

We next interrogated the plasma-level variability for the 42 proteins whose clinical assays have been approved by FDA (Anderson, 2010) and that were quantified in the dataset. We observed that generally, their overall variability was lower than that of the other proteins across the cohort (*P *=* *0.0001, Figs4F and 2D). We partially ascribe the lower variability of clinically assayed proteins to their higher heritability (*P *=* *0.0133, Supplementary Fig S8) and to the fact that it is desirable for biomarkers to have relatively stable expression levels. Strikingly, we found that the plasma levels of a few reported biomarkers are strongly and preferably affected by a longitudinal effect (> 30%) compared to the heritability and environmental components (Fig4G). These include biomarkers whose measurements have been already cleared or approved by FDA (e.g. biotinidase (Kang *et al*, 2010), platelet basic protein, thyroxine-binding globulin, etc.) and candidate proteins not yet cleared. For example, triosephosphate isomerase (TPIS) was discovered as a promising blood biomarker for metastatic non-small cell lung cancer (Patel *et al*, 2011), especially lung squamous cell carcinoma (Zhang *et al*, 2009). 14-3-3 protein zeta/delta (1433Z) was discovered as a putative prognostic marker for renal cell carcinoma (Masui *et al*, 2013) and for monitoring chemotherapy in breast cancer (Hodgkinson *et al*, 2012). And plasma level of soluble CD14 was indicated to be an independent predictor for human immunodeficiency virus infection. Our data therefore indicate that for the application of these proteins as predictive biomarkers, the longitudinal factors (such as aging) dependent variability should be considered. The incorporation of variability over time with stochastic variability of protein biomarkers will enable the refinement of risk scores estimated by such biomarkers.

### Identification of *cis-*protein quantitative trait loci *(*pQTL) influencing plasma protein levels

**Figure 5:**
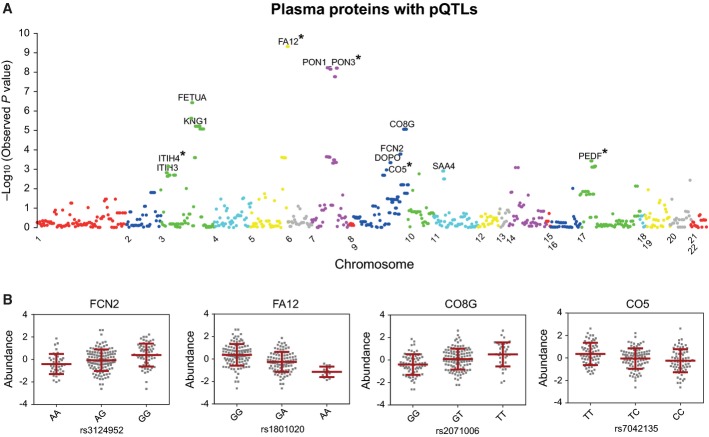
pQTL discovery in human plasma A Manhattan plot of the best *P*-value per gene, highlighting the 13 statistically significant pQTL associations. The asterisks indicate that the corresponding eQTLs were found in human tissues. The cutoff of the *P*-value is 6.166e-3.

Finally, we carried out association analysis to identify *cis-*SNPs regulating the levels of 303 (of the 342 measured) proteins that we could uniquely map to known genes. We first selected a total of 113 out of 116 individuals which passed the genotyping data quality control step. Second, we used the twin proteomic data to map the protein quantitative trait loci, or pQTLs by determining the statistical significance of the association between SNPs 1 Kb up and down the transcription start site for each gene and protein expression values. The final number of tested SNPs was 758. We found 13 genes with at least a statistically significant pQTL (Fig5A). Among them, four plasma proteins (ficolin-2, coagulation factor XII, complement component C8 gamma chain and complement C5) were annotated with the “innate immune response” process. The close association between genotype alleles and protein levels is shown in Fig5B. Similar distributions were obtained for all identified pQTLs (Supplementary Fig S9). We observed that most of the discovered pQTLs lie in regulatory regions and only 2 of them are in the coding region, but synonymous (Supplementary Table S4). To explore the functional role of the pQTLs, we assessed whether they have an effect on gene expression. We checked the association of the pQTLs with gene expression in four tissues (fat, skin, blood and lymphocyte cell lines (LCL)) in a cohort of about 800 female twins of the same population that are part of the Twin UK cohort with an identical age range. We called gene expression QTLs (eQTLs) in the four tissues by using gene expression measured by RNA-seq and genotype information (Buil *et al*, 2015). We could measure the gene expression of 9 of the 13 proteins with pQTLs, and we found that 5 of the 9 pQTLs are associated with eQTLs in at least one of the four tissues (Fig5A and Supplementary Table S4).

B Examples of pQTLs: plasma protein levels among the cohort of four proteins associated with innate immune response distributed by distinct genotypes of the SNPs (see Supplementary Table S3 for all abbreviations of protein names).

We also compared our pQTLs to those published previously by Johansson *et al* (2013) which were not significant in our sample. This fact might be partially explained by the distinctive sample cohorts used. To further investigate if the difference in detection was just a matter of power, we checked at the *P*-values of Johansson's pQTLs in our study. If the Johanson's pQTLs were present in our sample, even if they were not statistically significant, we would expect to see an enrichment of small *P*-values in our sample. We estimated the mean of the *P*-value of the Johanson's pQTL associations in our sample, and compared this mean value with the distribution of means expected if there were no signal in our sample. Supplementary Fig S10 shows the expected distribution of mean values under the null hypothesis (no signal of Johansson's pQTLs in our sample) and the actual observed mean. From there, we calculated a *P*-value equals 0.0035 that supports the notion that with a larger sample, we would find some of the pQTLs described by Johansson *et al* (2013).

To estimate the relative contribution of the pQTLs to protein variability, we estimated the proportion of protein variance explained by each pQTL. We observed that these pQTLs explained between 3 and 19% of the protein's variance with an average of about 8.5%. To compensate the fact that heritability might be not well separated with the estimates of common environment, we then estimated the contribution of the pQTL to the total family component consisting of both heritability and common environment component. We observed that pQTLs explain between 6 and 68% of the family component, with an average of about 25% (Supplementary Table S5). Finally, we used the online annotation tool SnpNexus (Dayem Ullah *et al*, 2012) to annotate the pQTLs as disease SNPs. We found that two of the pQTLs are associated with the following diseases: rs1801020 on gene *F12* with cardiovascular disease (Santamaria *et al*, 2004; Cochery-Nouvellon *et al*, 2007) and rs2071042 on gene *ITIH4* with hypercholesterolemia (Fujita *et al*, 2004). In summary, we found pQTLs for 13 proteins that explained about 8.5% of the protein-level variance.

## Discussion

The variability of human proteins within a population lays a basal-level foundation for relating protein expression patterns to disease (Nedelkov *et al*, 2005). The knowledge of the protein variability therefore stands to have an immediate impact in the field of clinical proteomics. In this study, we present the first study that addresses the degree of human protein variability and its origins using a quantitatively accurate proteomic approach and the rigorously controlled biological samples. Specifically, we used SWATH-MS (Gillet *et al*, 2012) to robustly and systematically dissect the generic and genetic variance within a cohort of 232 healthy twins by focusing on the proteome of the most intensely studied clinical samples, the human plasma. This analysis has become possible by the development of new mass spectrometric techniques that generate quantitatively accurate and highly reproducible datasets across large sample cohorts. Herein, by combining the extensive shotgun analysis presented in this study and the recently published SWATH-MS assay compendium for the human proteome (Rosenberger *et al*, 2014), we also configured the to-date most comprehensive spectral library for quantifying 1,667 human plasma proteins. We now provide this high-quality spectral library as a transferrable resource deposited in ProteomeXchange, which can be easily downloaded as a stand-alone MS assay repository to support those future targeted proteomic studies focused on the human plasma. Using this library and a targeted data analysis strategy, we identified on average 425 plasma proteins in each individual. This number is 3.3 times higher than that reported in the previous large-scale plasma proteomic survey by Johansson *et al* (2013). Further, in comparison to an extensive SRM analysis of body fluids that targeted more than 1,000 cancer-associated proteins (CAPs) and detected 73 proteins in the crude plasma digest (Huttenhain *et al*, 2012), herein we identified 99 CAPs and these were quantified across the whole twin cohort. Besides the high proteome coverage achieved, we consistently quantified 342 unique plasma proteins (assigned by 1,904 informative peptides) among the whole cohort with a highly completed data matrix facilitated by OpenSWATH framework (Rost *et al*, 2014). Most importantly, the technical variability of our SWATH-MS profiling was indeed extremely low (an average CV of 7.2%), which, in essence, significantly reduced the technical variance and allowed for the rigorous decomposition of biological variances that can be relatively small.

In our longitudinal twin strategy, we used samples from twins at an older age (an average of 57.8 and 63.1 at two donations) for the practical consideration that many systematic diseases such as cancer occur more frequently at this age range. For example, over half (53%) of all cancers are diagnosed in adults aged 50–74 and over a third (36%) of all cancers are diagnosed in the elderly aged > 75 in UK, 2009–2011. Our biological variability analysis at this similar age range is thus beneficial to the further understanding of the plasma protein profiles reported from those biomarker discovery studies focusing on such diseases. Females were chosen to simplify the experimental design by excluding the gender variance component in the dissection model due to the limited sample size.

The same trait measured over an individual's lifetime can have different genetic effects influencing it (Visscher *et al*, 2008). Therefore, compared to a parent–children-based approach (Johansson *et al*, 2013) which normally needs to remove the aging effect as a confounding factor, twin studies based on the longitudinal sampling of the same individuals are preferable to uncover the traits specifically regulated during a temporal period. We herein employed relatively longer longitudinal intervals of sampling, so that the controlling mechanism of plasma dynamics along with aging process during an average of 5.2 years could be investigated. We found the 5-year span was long enough to cause significant quantitative variability for 17.5% (that is, 60 out of 342) of plasma proteins. For slowly progressing diseases such as prostate cancer, 5 years might be not long enough to reveal the whole disease process. However, for many other general diseases, a span of 5 years is clinically relevant. For example, for ovarian cancer, the serum biomarkers were reported to provide evidence of the cancer 3 years before clinical diagnosis (Anderson *et al*, 2010). Also for diabetic nephropathy, the urinary levels of collagen fragments were demonstrated as prominent biomarkers 3–5 years before onset of macro-albuminuria (Zurbig *et al*, 2012). Previously, a 4-year difference in long-term storage was tested with no effect on plasma protein levels (Mitchell *et al*, 2005). This is consistent with our result where decreased genetic control along with the 5-year span can be successfully revealed. The biological confounding factors along with the 5-year span could include cancer, diabetes and other diseases developed, a change in medications consumed and menopausal status in elder women, as listed in Supplementary Table S1 as well as other temporal factors of unknown origin. With the limited cohort size, we could not dissect the variance contributed by these factors from the aging process in the present study. Of note, the relative longitudinal process explained much more variance than the real age of each individual (Supplementary Fig S7), demonstrating the necessity of the longitudinal strategy applied on the same individuals. With higher sample throughput rendered by the fast-developing proteomic instruments and workflows, a further, larger scale twin survey involving multiple longitudinal sampling points, a longer term age span that extends the age range investigated and both genders, would be ideal in the future to reinforce the understanding of genetic and longitudinal regulations of plasma protein expressions.

Additionally, the pQTLs we observed in the cohort may explain just a fraction of the genetic effects that control protein level. Other genetic variants in the *cis-*region around the functional gene plus genetic variants in *trans-*, far from the gene, are expected to explain the remainder of the observed heritability. The identification of these variants would require much larger sample sizes.

Moreover, we did not adopt the affinity depletion or enrichment approaches (which may bring more protein identifications) except for the step of spectral library generation, so that protein-dependent technical variation between individuals in the sample preparation step can be minimized to increase the power for our proteomic variance investigations. For future comparative studies, the relative variability derived from this study for certain plasma proteins (e.g. those interacting with albumin) might need to be adjusted by factoring in the technical variations, for example, those from immunodepletion (Dayon *et al*, 2014) or protein isolation steps, if indeed these steps are used.

Until now, biomarker discovery studies have almost entirely been focused on the comparison of the protein levels between disease and normal cases and were severely limited in sample size. However, the inherent (in)stability of proteins is biologically associated with its genetic architecture and thus, resultant confounding factors might obscure the biomarker analyses. Here, we show that the roles of the heritable, environmental and longitudinal determinants in controlling plasma protein levels are different for different proteins and functional clusters, and we noted that longitudinal effects might decrease the genetic control of protein levels and reduce the regulation power in controlling the variability of high abundant plasma proteins. All such valuable information will increase the ability of future studies to assign statistical significance to potential protein biomarkers.

Understanding the underlying genetic determinants of biomarkers can be useful in diagnosis or prognosis of diseases in two different ways. First, for biomarkers known to be causal for a disease, genetic variants associated with a biomarker become themselves genetic biomarkers for the disease. In this case, the quality of the genetic biomarker will depend on the association with the original biomarker. Second, for biomarkers that are not causal for a disease, the information about the disease that is carried by the biomarker does not come from itself but from unknown marker associated with, and causal for, the diseases. In this case, removing the effect of genetic variants that affect the biomarker abundance should increase the relative information of the unknown causal marker, making the biomarker more informative, that is, more associated with the disease. As examples, two identified plasma proteins, lipoprotein A (LPA) and C-reactive protein (CRP), were previously established as biomarkers for indicating coronary artery disease (CAD) risk (Jansen *et al*, 2014). LPA was reported as a causal risk factor for CAD (Jansen *et al*, 2014), and pQTLs for LPA are also associated with CAD (Kamstrup *et al*, 2009). That means that these pQTLs could be genetic biomarkers for CAD. On the other hand, pQTLs for CRP showed no association with CAD (Elliott *et al*, 2009); in this case, removing the effect of the genetic variants associated would result in a better scrutiny of CRP as a CAD biomarker.

In conclusion, the large-scale establishment of the heritability dimension of plasma proteins (or other subproteomes of human, or even the entire human proteome in the future) and the identification of pQTLs explaining a fraction of protein-level variation will sharpen our understanding of protein expressions, functions and temporal dynamics from the heritability perspective and aid the more efficient biomarker discovery. Our study serves as a first step in understanding the population-level variance components and eventually incorporating them in disease risk assessment.

## Materials and Methods

### Sample recruitment and collection

A total of 116 twins, comprising 22 DZ and 36 MZ pairs, were ascertained from the Twins UK Adult Twin Registry at King's College London of approximately 11,000 twins (http://www.twinsUK.ac.uk) (Moayyeri *et al*, 2013) and invited to participate in this study. Eligible twins were healthy, Caucasian females of north European descent and aged between 38 and 78 years. They provided written informed consent. Zygosities were confirmed by genotype. We applied a longitudinal twin strategy to recruit samples. These healthy twins were selected at an average age of 57.8, and the time intervals between two donations range from 678 to 2,718 days, with a mean of 1,910 days (i.e. 5.2 years). This study was approved by St. Thomas' Hospital Research Ethics Committee. The twins have been shown to be generalizable to the general singleton population (Andrew *et al*, 2001). Fasting blood samples were collected at all visits under identical conditions (all twins fasted overnight for 10 h before the scheduled visits). Plasma was obtained from each sample by centrifuging at 2,000 *g* for 10 min at room temperature, aliquoted and instantly stored at −80°C. Complete Protease Inhibitor Cocktail (Roche) was added upon thawing. None of the samples were thawed more than twice before analysis.

### Sample preparation and protein digestion

Crude plasma samples were centrifuged at 18,400 *g* for 10 min at 4°C. The following sample preparation steps were performed with 96-well format plates with five whole-process experimental replicates distributed in different plates. 5 μl of plasma from each sample was diluted to 50 μl and filtered by G-10 gel filtration cartridges (Nest Group Inc.). Three external proteins were spiked (bovine alpha-1-acid glycoprotein with the targeted plasma level at 85 μg/ml, bovine fetuin-B at 8.5 μg/ml and human prostate-specific antigen at 0.85 μg/ml) before 80 μl of 10 M urea in 100 mM ammonium bicarbonate was added into each sample for denaturing at 37°C, 30 min. After reduction and alkylation with 10 mM tris(carboxyethyl)phosphine (Sigma-Aldrich) and 20 mM iodoacetamide (Sigma-Aldrich), the samples were diluted to 1 M urea and were digested with sequencing-grade porcine trypsin (Promega) at a protease/protein ratio of 1:50 overnight at 37°C (Huttenhain *et al*, 2012). Digests were purified with Vydac C18 Silica MicroSpin columns (The Nest Group Inc.). An aliquot of retention time calibration peptides from iRT-Kit (Biognosys) was spiked into each sample at a ratio of 1:30 (v/v) before all LC-MS analysis, to correct relative retention time differences between runs (Escher *et al*, 2012). Selected, heavy isotope-labeled internal standard peptides according to our previous study were synthesized (Huttenhain *et al*, 2012) (JPT Peptide Technologies and Thermo Fisher) and spiked into each sample for SRM and SWATH-MS measurements.

### Plasma depletion and SAX fractionation

For the comprehensive shotgun analysis, crude plasma mixture from all the samples was firstly depleted of the 14 most abundant proteins with the multiple affinity removal system (MARS Hu-14 spin cartridge; Agilent Technologies) according to the manufacturer's instruction. Depleted samples were exchanged with Vivaspin 500 concentrators with a 5,000 molecular weight cutoff (Sartorius Stedim Biotech), denatured in 6 M urea and then diluted and digested with trypsin as above (Huttenhain *et al*, 2012). 50 μg of the resulting peptides was then separated into six fractions by strong anion exchanger (SAX) and purified on C18 StageTips as previously described (Wisniewski *et al*, 2009). The depleted sample was also directly digested and analyzed without SAX fractionation.

### Shotgun proteomic measurement

The depleted and fractionated peptides were all measured by an AB Sciex 5600 TripleTOF mass spectrometer operated in data-dependent acquisition (DDA) mode. The mass spectrometer was interfaced with an Eksigent NanoLC Ultra 2D Plus HPLC system as previously described (Gillet *et al*, 2012; Collins *et al*, 2013; Liu *et al*, 2013b). Peptides were directly injected onto a 20-cm PicoFrit emitter (New Objective, self-packed to 20 cm with Magic C18 AQ 3-μm 200-Å material) and then separated using a 120-min gradient from 2 to 35% (buffer A 0.1% (v/v) formic acid, 2% (v/v) acetonitrile, buffer B 0.1% (v/v) formic acid, 90% (v/v) acetonitrile) at a flow rate of 300 nl/min. MS1 spectra were collected in the range 360–1,460 m/z. The 20 most intense precursors with charge state 2–5 which exceeded 250 counts per second were selected for fragmentation, and MS2 spectra were collected in the range 50–2,000 m/z for 100 ms. The precursor ions were dynamically excluded from reselection for 20 s.

### SWATH-MS measurement

SWATH-MS measurements were performed with peptide mixtures generated by digesting crude plasma samples. The unfractionated, total peptide samples were analyzed to minimize confounding factors introduced by sample handling. The same LC-MS/MS system used for DDA measurements was used for SWATH analysis (Gillet *et al*, 2012; Collins *et al*, 2013; Liu *et al*, 2013b). Specifically, in SWATH-MS mode, the instrument was specifically tuned to optimize the quadrupole settings for the selection of 25-m/z wide precursor ion selection windows. Using an isolation width of 26 m/z (containing 1 m/z for the window overlap), a set of 32 overlapping windows was constructed, covering the precursor mass range of 400–1,200 m/z. The effective isolation windows can be considered as being 399.5–424.5, 424.5–449.5, etc. SWATH-MS2 spectra were collected from 100 to 2,000 m/z. The collision energy (CE) was optimized for each window according to the calculation for a charge 2+ ion centered upon the window with a spread of 15 eV. An accumulation time (dwell time) of 100 ms was used for all fragment ion scans in high-sensitivity mode, and for each SWATH-MS cycle, a survey scan in high-resolution mode was also acquired for 100 ms, resulting in a duty cycle of ∽3.4 s. Six repeated SWATH-MS measurements were performed on one of the samples to access the technical variability.

### SRM measurement

For SRM analysis, peptide samples were analyzed on a hybrid triple quadrupole/ion trap mass spectrometer (5500QTRAP, AB Sciex) equipped with a nanoelectrospray ion source. Chromatographic separation of peptides was performed by a nanoLC ultra 1Dplus system (Eksigent) coupled to a 15-cm-fused silica emitter. Peptides were separated in a 35-min gradient of 5–35% acetonitrile in 0.1% formic acid (v/v) at a flow rate of 300 nl/min (Huttenhain *et al*, 2012; Liu *et al*, 2013b). Both Q1 and Q3 operated at unit resolution and a cycle time of 3 s at scheduled mode (4 min window). CEs were calculated according to previous studies (Lange *et al*, 2008; Liu *et al*, 2013b). SRM data were analyzed using Skyline (MacLean *et al*, 2010) and normalized based on the external proteins spiked and the heavy peptide standards.

### Spectral library generation for SWATH-MS

Profile-mode.wiff files from shotgun data acquisition were centroided and converted to mzML format using the AB Sciex Data Converter v.1.3 and converted to mzXML format using MSConvert v.3.04.238. The MS2 spectra were queried against the reviewed canonical Swiss-Prot complete proteome database for human (Nov. 2012) appended with common contaminants and reversed sequence decoys (Elias & Gygi, 2007) (40,951 protein sequences including decoys). The SEQUEST database search (Yates *et al*, 1995) through Sorcerer PE version 4.2 included the following criteria: static modifications of 57.02146 Da for cysteines and variable modifications of 15.99491 Da for methionine oxidations. The parent mass tolerance was set to be 50 p.p.m, and mono-isotopic fragment mass tolerance was 0.5 Da (which was further filtered to be < 0.05 Da for building spectral library); semi-tryptic peptides and peptides with up to two missed cleavages were allowed. The identified peptides were processed and analyzed through Trans-Proteomic Pipeline 4.5.2 (TPP) (Keller *et al*, 2005) and were validated using the *PeptideProphet* score (Keller *et al*, 2002). All the peptides were filtered at a false discovery rate (FDR) of 1%. The raw spectral libraries were generated from all valid peptide spectrum matches and then refined into the non-redundant consensus libraries (Collins *et al*, 2013) using SpectraST (Lam *et al*, 2007). For each peptide, the retention time was mapped into the iRT space (Escher *et al*, 2012) with reference to a linear calibration constructed for each shotgun run, as previously described (Collins *et al*, 2013). The MS assays, constructed from Top six most intense transitions with Q1 range from 400 to 1,200 m/z excluding the precursor SWATH window, were used for targeted data analysis of SWATH maps.

A published high-confidence list of 1,929 human plasma proteins complied from PeptideAtlas (Farrah *et al*, 2011) was merged with an in-house compendium of MS assays for the targeted detection of any of more than 10,000 human proteins in SWATH-MS datasets (Rosenberger *et al*, 2014), and correspondingly, the highly specific MS assays of 1,550 plasma proteins were extracted. The spectral library from our shotgun analysis was then combined with the library of these 1,550 proteins at the peptide level, whereas the assays coming from the latter case were only accepted if the corresponding peptides were not identified by our shotgun effort.

### Targeted data analysis for SWATH maps

SWATH-MS.wiff files were first converted to profile mzXML using ProteoWizard (Kessner *et al*, 2008). The whole process of SWATH-targeted data analysis was carried out using OpenSWATH (Rost *et al*, 2014) running on an internal computing cluster. OpenSWATH utilizes a target-decoy scoring system like mProphet to estimate the identification of FDR (Rost *et al*, 2014). The best scoring classifier that was built from the sample of most protein identifications was utilized in this study. Based on our final spectral library for human plasma proteome, OpenSWATH firstly identified the peak groups from all individual SWATH maps at a global peptide FDR = 1% (enabled by the strict FDR cutoff of 0.0307% at the level of total peak groups) and aligned them between SWATH maps based on the clustering behaviors of retention time in each run with a non-linear alignment algorithm (Weisser *et al*, 2013). Specifically, only those peptide peak groups identified in more than 1/3 samples were reported and considered for alignment with the max FDR quality of 0.1 (quality cutoff to still consider a feature for alignment) and/or the further constraint of < 100 s RT difference in LC gradient after iRT normalization.

Next, to obtain a high-quality quantitative data at the protein level, at the first step, we only accepted those proteins whose peptides had been identified in at least 90% of all the samples for proteomic profiling. Moreover, peptides that were shared between different proteins [non-proteotypic peptides (Mallick *et al*, 2007)] were discarded for quantification. The re-quantification option by OpenSWATH (Rost *et al*, 2014) was then enabled to re-quantify the missing values. Secondly, to retrieve the protein quantification information from those peptides identified in more than 1/3 but < 90% of the samples, we firstly enabled the re-quantification option of OpenSWATH to re-quantify the missing values by the local MS2 noise, then we fit our model at peptide-level, accepted those peptides whose residual variance (see below section of variance decomposition) was < 65% (so that an equal average of residual variance was achieved when compared to the first step). To quantify the protein abundance levels across 232 samples, we summed up the most abundant identified peptides (that is, 595 peptides) for each protein (Top three peptides, if > 3 peptides identified). This allows for reliably estimating global protein-level changes as shown in previous studies (Cima *et al*, 2011; Ludwig *et al*, 2012; Liu *et al*, 2013b, 2014; Weisser *et al*, 2013). The re-quantification in both steps totally retrieved signals for 10.07% of the missing cells in the protein-level data matrix. The two-step filtering strategy essentially discarded those peptides that were not detected in majority of the samples (but got imputed by highly variable noisy background using re-quantificaton algorithm) and therefore filtered the consistent, high-quality protein signal groups among samples so that enough data points could be used for further variance decomposition. In total, quantification data of 342 unique Swiss-Prot proteins across all the 232 twin plasma samples were used for subsequent analyses.

### Genotyping and imputation

Our samples are a subset of the Twins UK dataset. SNP genotyping of the Twins UK dataset (*N* = 6,000) was done with a combination of Illumina HumanHap300, HumanHap610Q and 1M-Duo chips and was performed by The Wellcome Trust Sanger Institute and National Eye Institute via NIH/CIDR. Similar exclusion criteria were applied to each of the three datasets separately. Sample exclusion criteria were (i) sample call rate < 98%, (ii) heterozygosity across all SNPs ≥ 2 SD from the sample mean, (iii) evidence of non-European ancestry as assessed by PCA comparison with HapMap3 populations and (iv) observed pairwise IBD probabilities suggestive of sample identity errors. We corrected misclassified monozygotic and dizygotic twins based on IBD probabilities. SNPs exclusion criteria were: (i) Hardy–Weinberg *P*-value < 10^−6^, assessed in a set of unrelated samples; (ii) MAF < 1%, assessed in a set of unrelated samples; and (iii) SNP call rate < 97% (SNPs with MAF ≥ 5%) or < 99% (for 1% ≤ MAF < 5%). Prior to merging, we performed pairwise comparison among the three datasets and further excluded SNPs and samples to avoid spurious genotyping effects, identified as follows: (i) concordance at duplicate samples < 1%; (ii) concordance at duplicate SNPs < 1%; (iii) visual inspection of QQ plots for logistic regression applied to all pairwise dataset comparisons; (iv) Hardy–Weinberg *P*-value < 10^−6^, assessed in a set of unrelated samples; and (v) observed pairwise IBD probabilities suggestive of sample identity errors. We then merged the three datasets, keeping individuals typed at the largest number of SNPs when an individual was typed at two different arrays. Samples were genotyped on a combination of the HumanHap300, HumanHap610Q, 1M-Duo and 1.2MDuo 1M Illumina arrays. Samples were imputed into the 1,000 Genomes Phase 1 reference panel (data freeze, 10/11/2010) (Abecasis *et al*, 2012) using IMPUTE2 (Howie *et al*, 2009) and filtered [MAF < 0.01, IMPUTE info value < 0.8 and HWE (*P* < 1e-5)]. We ended with 3,552,380 SNPs in 113 of our 116 individuals.

### Variance decomposition of protein and peptide level

It is known that variance components models are sensitive to traits that have a distribution different from normal. To get robust results, we transformed the protein quantifications using a rank normal transformation. To estimate the variance components of plasma protein level, we used a linear mixed model with two fixed effects and four random effects: 

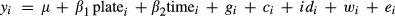


where plate and time are fixed effects representing the sample plate and the time interval between the two time points (time is 0 in time point 1 and the actual time interval in time point 2) and the rest are random effects:

*g* is the polygenic effect.
*c* is the shared environment effect.
*id* is the effect the individual environment.
*w* is effect of the visit (longitudinal effects, or aging effect in this study).
*e* are the residuals.

These data present a complex structure of correlation because we have related individuals measured twice. The expected correlation structure between a twin pair can be represented as: 

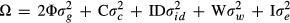


where 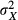 represent the variance of the X component:

2Φ is the expected kinship coefficient based on the observed relationships—that is, 1 for MZ twins, ½ for DZ twins and 0 otherwise.
C is the shared environment matrix, 1 per all the twin pairs.
ID is the individual matrix, it takes into account that the same individual is measured in time point 1 and in time point 2. It is 1 for samples of the same individual in time points 1 and 2, and 0 otherwise.
W is the visit matrix, it takes into account the fact that the two sisters of a twin pair went together to the visit. It is 1 for the two sisters at each visits, 0 otherwise.
I is the identity matrix.

Heritability was defined as 
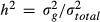
, and the rest of the variance components were defined in the same way. The statistical analyses were carried out using SOLAR v 6.5.8 software package (Almasy & Blangero, 1998).

It is worth to note that, due to the limited samples size, it is difficult to separate perfectly the heritability and the common environmental effects components. The sum of these two components interpreted as a family effect, however, estimates more robustly (Kato *et al*, 2011; Nicholson *et al*, 2011).

### pQTL determination

High-throughput experiments can generate batch effects that are difficult to control. In the transcriptomics field, it is widely acknowledged that removing the first principal components of the expression dataset removes unknown batch effects and increases the power to find eQTLs. We borrowed this technique, and before the pQTL analysis, we regressed out the first 10 principal components of the protein data. Since our data samples are twins, they are not independent observations and we need to take that into account in our models. We used the approach previously described (Aulchenko *et al*, 2007a) to keep the residuals of a mixed model that removed the effects of the family structure using the implementation in GenAbel R package (Aulchenko *et al*, 2007b). We then transformed those residuals using a rank normal transformation to avoid undesired outlier effects of the associations.

To identify pQTL associations, we performed a linear regression of the transformed residuals on all the SNPs in a 1-Kb window around the transcription start site for each gene and kept the best association per gene. To assign statistical significance of our associations, we run 20,000 permutations for each protein to estimate a null distribution. We shuffled the sample name in the protein data and repeated the association analysis keeping the best association for each permutation. Using this null distribution, we calculated an empirical *P*-value per each protein. This approach is better than having a uniform threshold for all the proteins because we take into account that for some proteins, we test more SNPs than for others and then, the chances for spurious association are larger too. We performed the SNP significance analysis separately for time points 1 and 2 and then combined the *P*-values from the two time points using the Fisher method for those SNPs influencing protein abundance in the same direction. In this way, we got a single *P*-value per each protein. Finally, we call significant pQTLs based on a 10% FDR using the R package *q*-value (Storey & Tibshirani, 2003).

### Overlap with eQTLs

To check if the effect of our pQTLs on protein level was due to a change in gene expression, we looked for associations of the pQTL SNPs with gene expression measured using RNAseq technology in four tissues (fat, LCLs, skin and blood) in about 800 female twins (Buil *et al*, 2015). We analyzed each tissue and each gene separately. For each gene, we used a linear model to estimate the association between the pQTL and a normalized expression of each exon. We kept the best *P*-value per gene and estimated its statistical significance by comparison with a null distribution obtained by permuting the gene expression labels 10,000 times.

### Other bioinformatic analyses

Hierarchical clustering analysis (HCA) was performed by Cluster 3.0 on the log-transformed, 2-dimensional-centered and normalized peptide intensities and visualized by Treeview. R software was used for plotting histograms. All the *P*-values indicating the significance of the data distribution difference were also reported by R using the Wilcoxon rank-sum test with continuity correction (the command is wilcox.test). The paired Wilcoxon test was used to compare the contribution of heritability and family component between two time points. The proteins significantly affected by the four biological components were filtered based on either a *P* < 0.01 or the fact that the corresponding component explains more than 25% of total variance. The annotation of biological pathways and functional processes was done using David bioinformatics resource (Huang da *et al*, 2009), where the enrichment analysis was performed by taking all the proteins in our human plasma spectral library as background. The HDL protein list was extracted based on consensus summary of published HDL studies (Shah *et al*, 2013), whereas the non-HDL protein list contains those proteins that have not been supported as HDL protein in either of the studies (Shah *et al*, 2013).

### Data availability

All the raw data of mass spectrometry measurements (SWATH-MS and shotgun), together with the input spectral library for human plasma proteome and OpenSWATH results can be freely downloaded from ProteomeXchange Consortium (http://proteomecentral.proteomexchange.org) via the PRIDE partner repository (Vizcaino *et al*, 2014) with the dataset identifier PXD001064. SNP genotyping data can be accessed with the permission of the TREC committee.

## Supporting Information

Supplementary Tables S1–S5

Review Process File
