# Supplementary material for: Quantitative variability of 342 plasma proteins in a human twin population
Source: Mol Syst Biol. 2015 Feb 4;11(2):786. doi: 10.15252/msb.20145728 (PMC4358658; doi:10.15252/msb.20145728)
Supplement: Supplementary file 1 [file msb0011-0786-sd1.pdf]

Supplementary figures

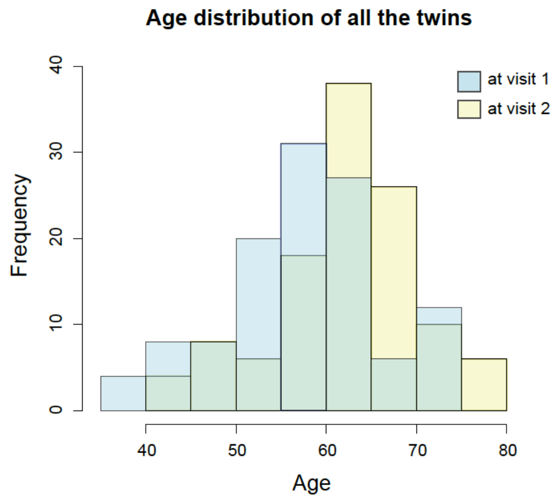

Figure S1. The age distribution of the twin samples at two visits.

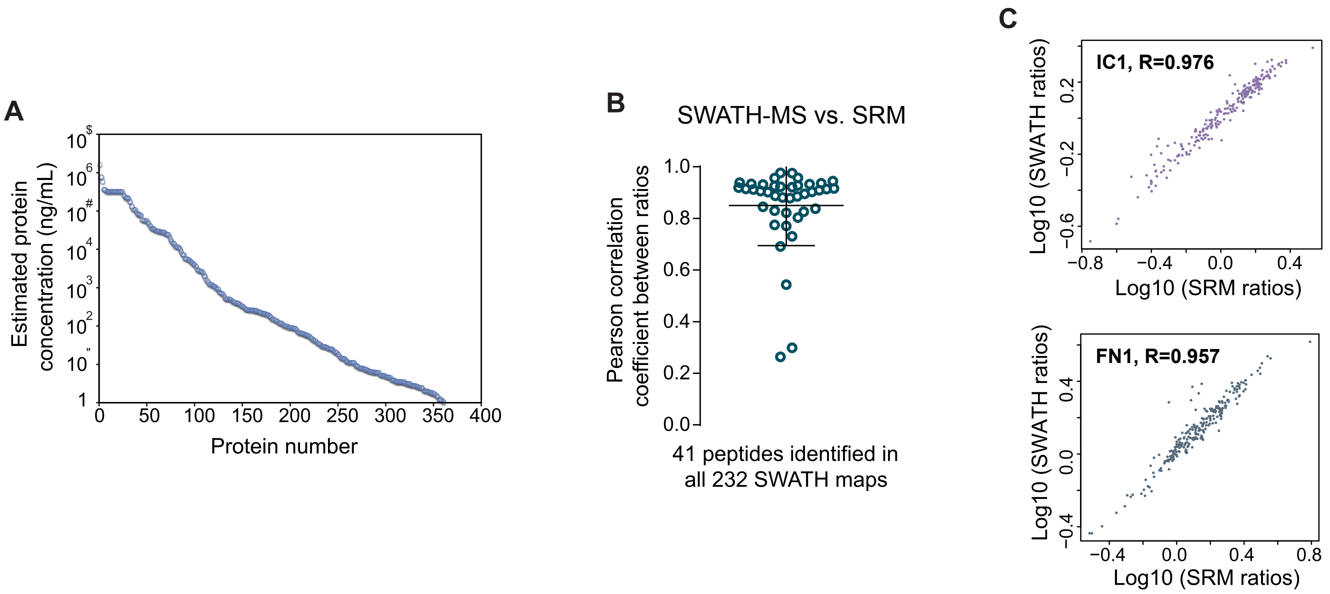

Figure S2. The analytical depth and reproducibility of SWATH-MS measurement.

A. Estimated protein concentrations for measured 534 plasma proteins in human blood were extracted from Human Plasma PeptideAtlas (PA) (www.peptideatlas.org/). Note that 361 out of 534 proteins have the estimated concentrations in PA, for which we suggest that our SWATH-MS technique identification and relative quantification of plasma proteins across six orders of magnitude (as low as several nanograms per milliliter for some proteins) among the cohort.

B. The ratios between light and heavy versions of 41 peptides detected in all the 232 SWATH maps were generally correlated well with SRM results among the samples, with a mean of R equals 0.85.

C. Two examples of Plasma protease C1 inhibitor (IC1) and Fibronectin (FN1) were illustrated, with best correlation between SWATH-MS and SRM to quantify the relative abundance among all 232 individuals.

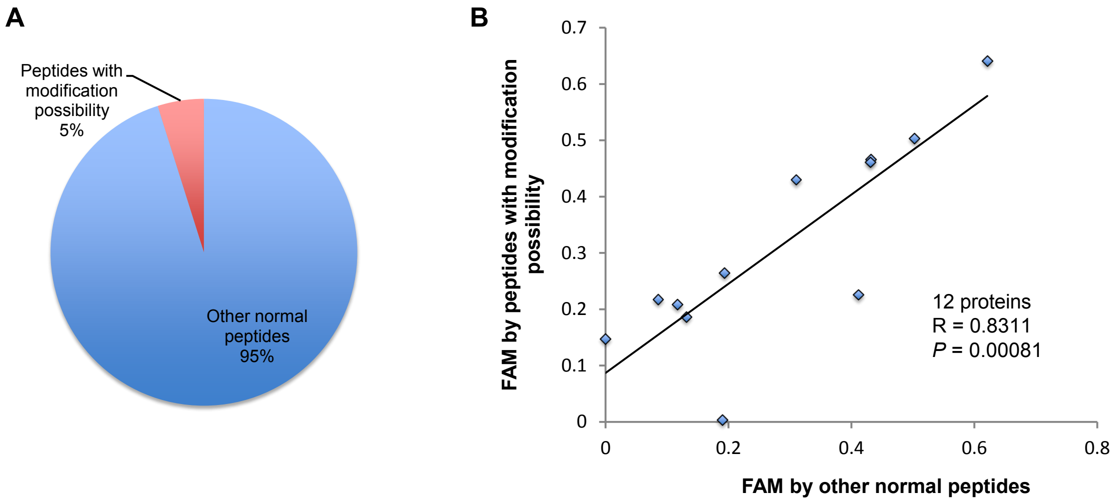

Figure S3. The technical bias due to potential peptide level modification has a negligible effect in the result of variance analysis.

A. Only 29 of 595 (i.e., <5% of) peptides that we included in the model have theoretical possibility of being modified, according to Swiss-Prot database.

B. The possibly modified peptides and normal peptides achieved the identical result in variance decomposition (P = 0.00081 for the significance of Pearson correlation), for those 12 proteins that has both peptide groups. FAM, family component variance (from the combination of heritability and shared environment).

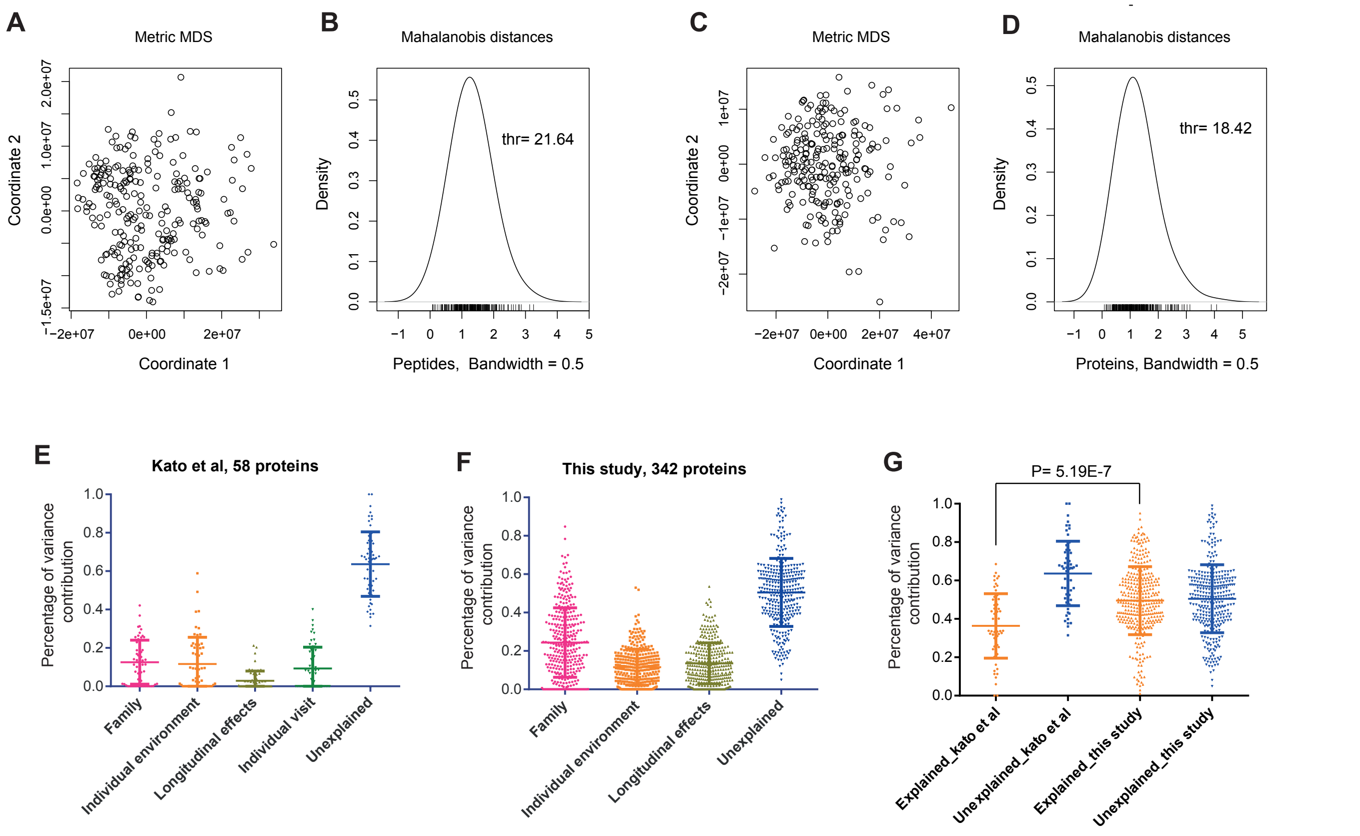

Figure S4. The SWATH-MS quantification robustness is translated into accurate estimation of different variance contributions.

Our data achieved a robust quantification of plasma proteome between 232 individuals, where no removal of outlier samples are needed. To investigate the presence of outlier samples in our study we projected the all quantified peptides **(A-B)** and their corresponding proteins **(C-D)** in two dimensions using multidimensional scaling (MDS) as implemented in the *cmdscale* command in R. As we can see from both peptide and protein levels, all the samples cluster together suggesting that there are no outliers in our samples. Assuming that the two MDS factors follow a bivariate normal distribution, the Mahalanobis distance from each point/sample to the origin should follow a chi-squared distribution with two degrees of freedom. The threshold to consider a sample as an outlier is 18.42 (critical value of 0.0001). Since all our samples have a distance much smaller that that, we confirmed that they are not outliers, in comparison to antibody based data from Kato et al (Kato et al, 2011), in which 2.2% samples are removed as outliers even after a specific probabilistic quotient normalization. **(E-F)** Compared to the antibody based quantification of Kato et al (Kato et al, 2011), our SWATH-MS yielded the heritability analysis for 284 more proteins; (**G)** Though we reported much more low-abundant proteins (many of them are of lower abundance compared to Kato et al (Kato et al, 2011)), a significant higher fraction of total phenotype variance that can be explained by biological stable factors in our data (*P*= 5.19e-7, Wilcoxon test).

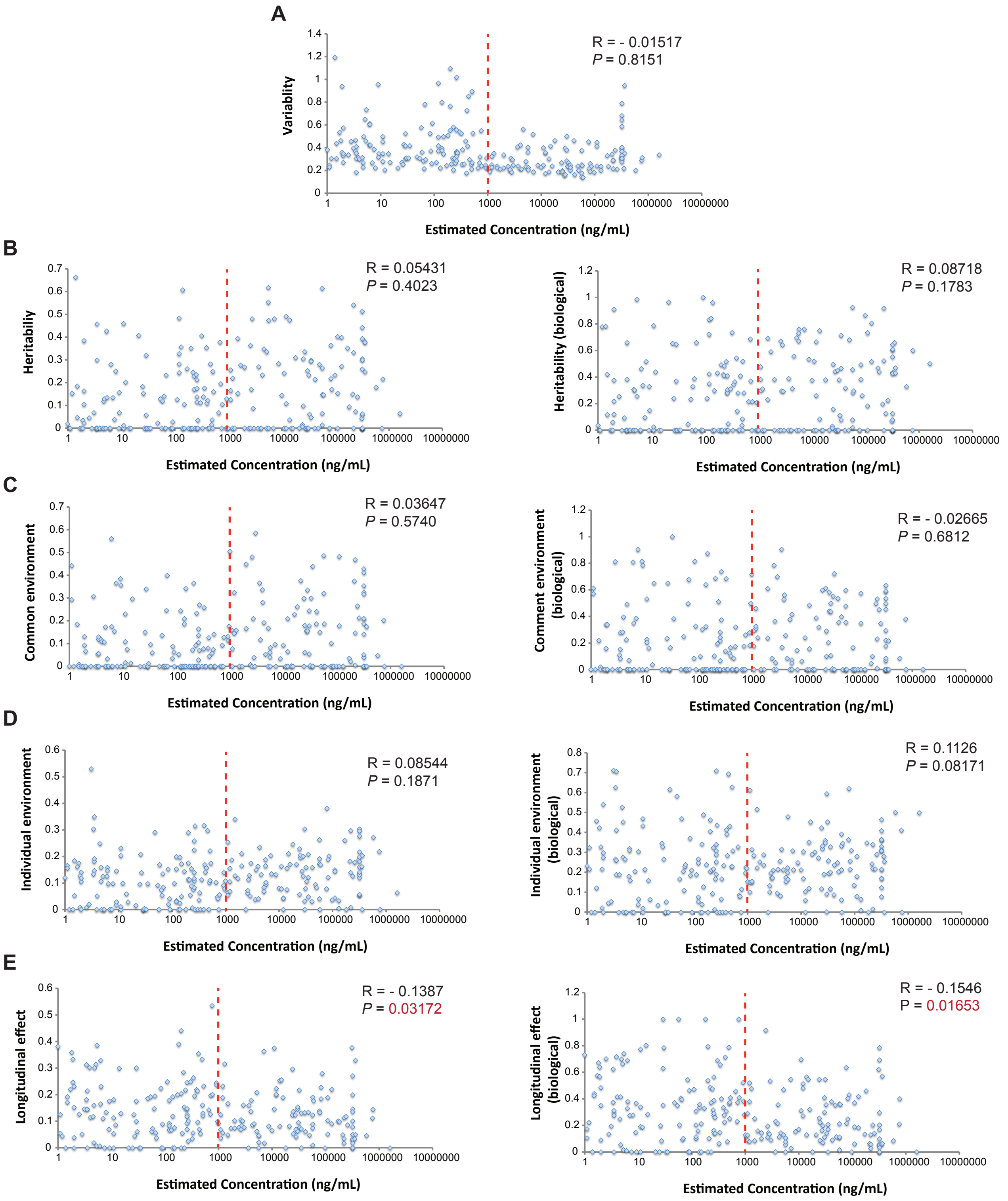

Figure S5. The biological variance dissection for plasma proteins of different concentrations. Note that we plot the protein variability and the determined contributions from heritability, shared environment, individual environment and longitudinal factors to the total variance and the total biological variance. The red dash line indicates the protein concentration of 1 μg/mL in human plasma. Note that the longitudinal factors seems to account for more variability for proteins whose concentration is below 1 μg/mL.

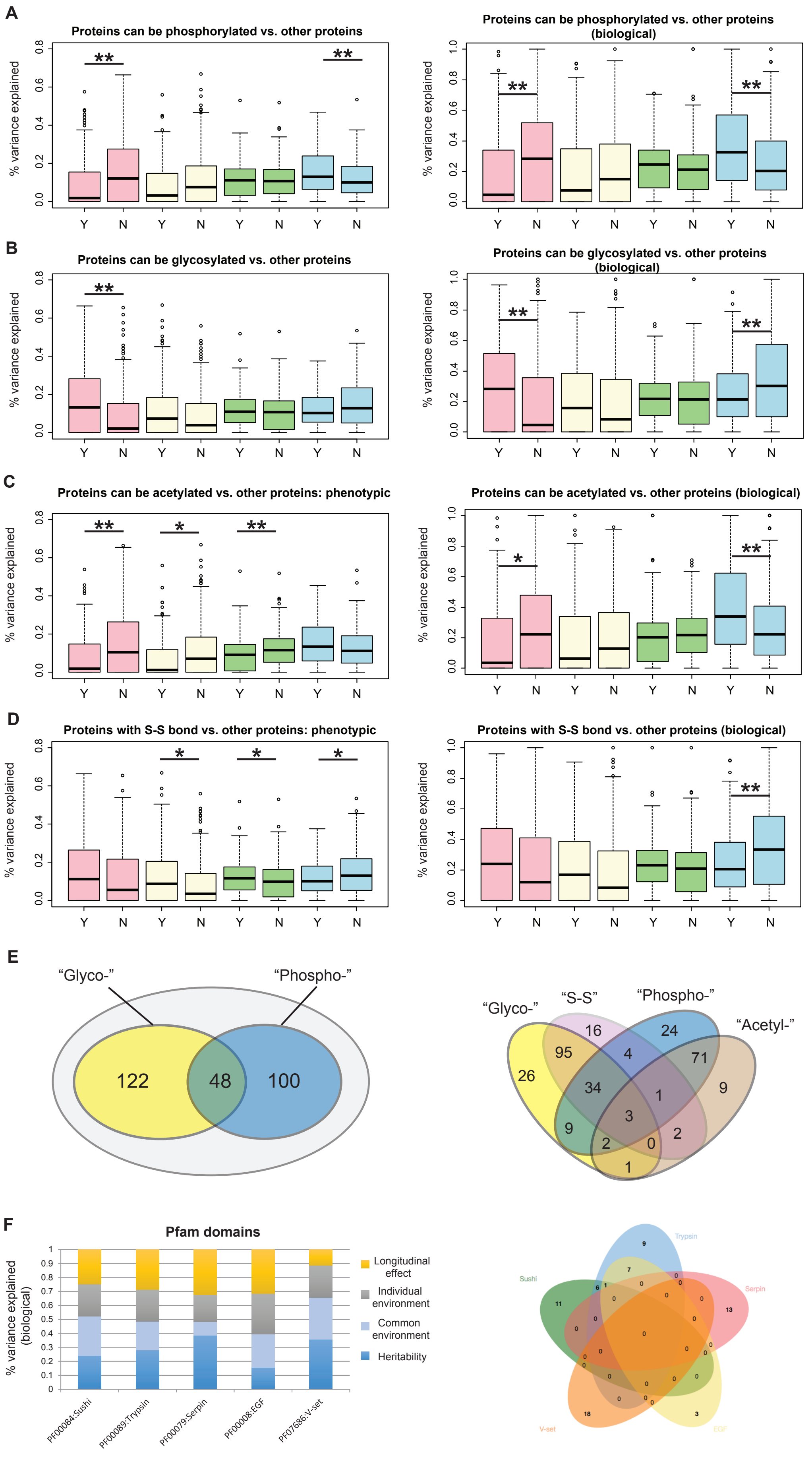
A
Proteins can be phosphorylated vs. other proteins
Proteins can be phosphorylated vs. other proteins (biological)
B
Proteins can be glycosylated vs. other proteins
Proteins can be glycosylated vs. other proteins (biological)
C
Proteins can be acetylated vs. other proteins: phenotypic
Proteins can be acetylated vs. other proteins (biological)
D
Proteins with S-S bond vs. other proteins: phenotypic
Proteins with S-S bond vs. other proteins (biological)
% variance explained
Y
N
**
*
E
"Glyco-"
"Phospho-"
122
48
100
"S-S"
"Acetyl-"
F
Pfam domains
% variance explained (biological)
Longitudinal effect
Individual environment
Common environment
Heritability
PF00084:Sushi
PF00089:Trypsin
PF00079:Serpin
PF00008:EGF
PF07686:V-set
Trypsin
Sushi
Serpin
V-set
EGF

Figure S6. The biological variance dissection for plasma proteins annotated with differential modifications and Pfam domains.

There are 170 proteins can be potentially glycosylated, 148 proteins might be phosphorylated, 89 proteins might be acetylated and 155 proteins could have disulfide bond according to Swiss-Prot database. The top five mostly identified Pfam domains are “Trypsin” (N=23 proteins), “V-set” (N=18), “Sushi” (N=18), “Serpin” (N=13) and “EGF” (N=11). **(A-D)** The comparison of variances explained by the four biological components (pink: heritability; light yellow: shared environment, green: individual environment), light blue: longitudinal factors) for proteins with potential modifications listed in Swiss-Prot. “Y” means the proteins are mapped to the corresponding modification, whereas “N” means other proteins without the specific modification listed. *, $P<0.05$ and **, $P<0.01$ indicate the statistical significances of the explained percentage of variances between “Y” and “N” groups. **(E)** The protein lists of potential modifications partially overlapped to each other. The annotated “glycoproteins” and the annotated “phosphoproteins” are relatively distinctive, while “glycoproteins” are highly overlapped with “disulfide bond” proteins whereas “phosphoproteins” are highly overlapped with “acetylated” proteins. **(F)** The lists of Pfam domains are relatively distinctive to each other. Because of the small number of each domain type annotated, we only plotted the average contributions of the four biological components for each of the five domain categories.

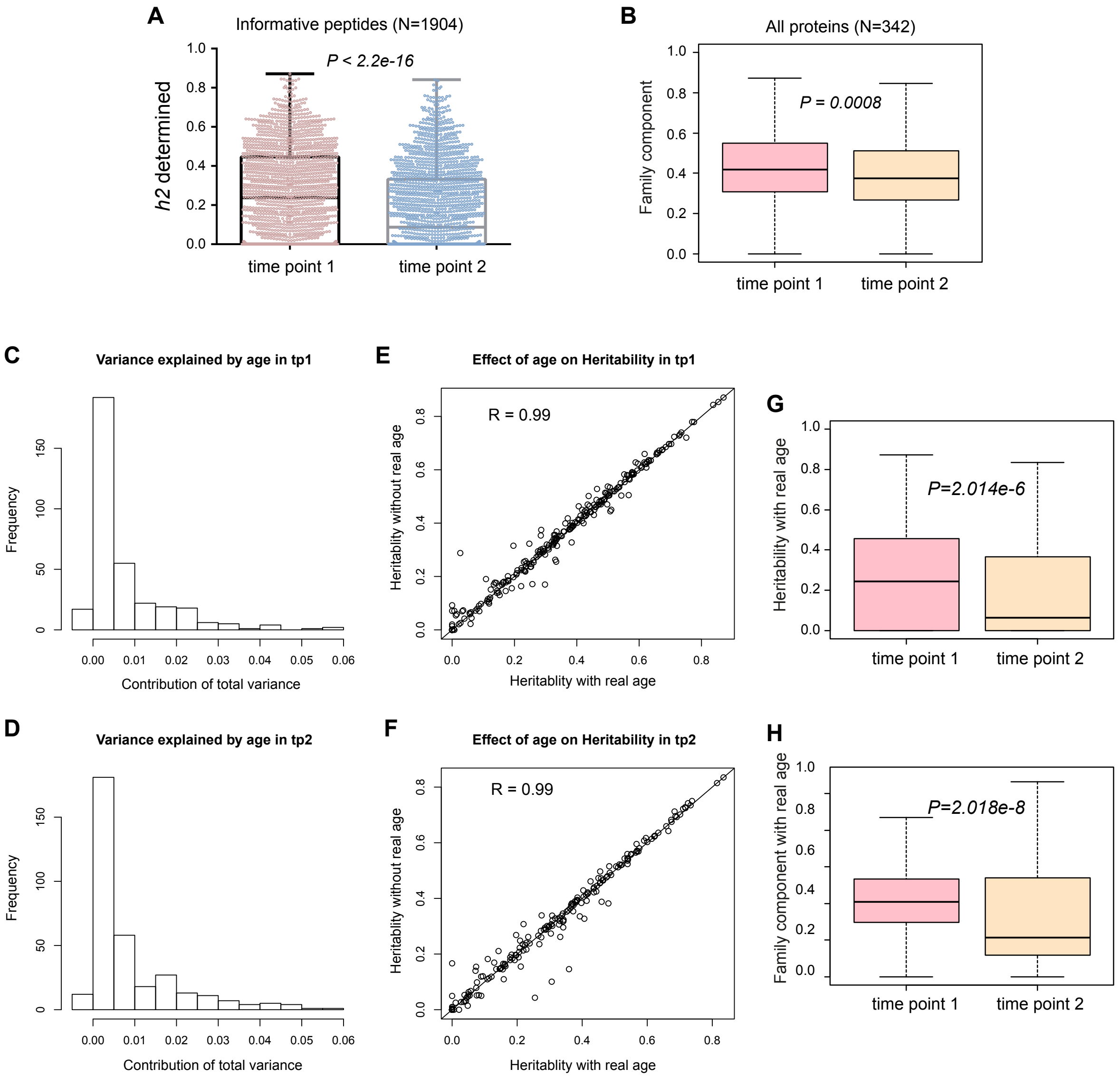

Figure S7. The real age incorporated in the variance model has only little effect in explaining total variance.

A. The decreasing trend of heritability control in plasma protein levels along with 5-year longitudinal process using quantitative data at the peptide- level. All the informative peptides (those peptides which are unique to the Swiss-Prot protein identification, but not only the high abundant ones for constituting protein data in Fig. 3e) were included.
B. The decreased trend of familial component contribution in explaining protein variability. The familiar component is the sum of the heritability and the common environment factor, whose percentage of contribution is decreased from time point 1 to time point 2.
C. The distribution of the contribution of the real age in the model for visit 1.
D. The distribution of the contribution of the real age in the model for visit 1.
E. The estimate of heritability is almost identical in the model with age and without age (correlation of 99%) at visit 1.
F. The identical heritability estimate at visit 2.
G. The heritability of plasma proteins corrected by real age effect showed the same decreasing trend between visits.
H. The family component contribution corrected by real age effect also showed the same decreasing trend between visits.

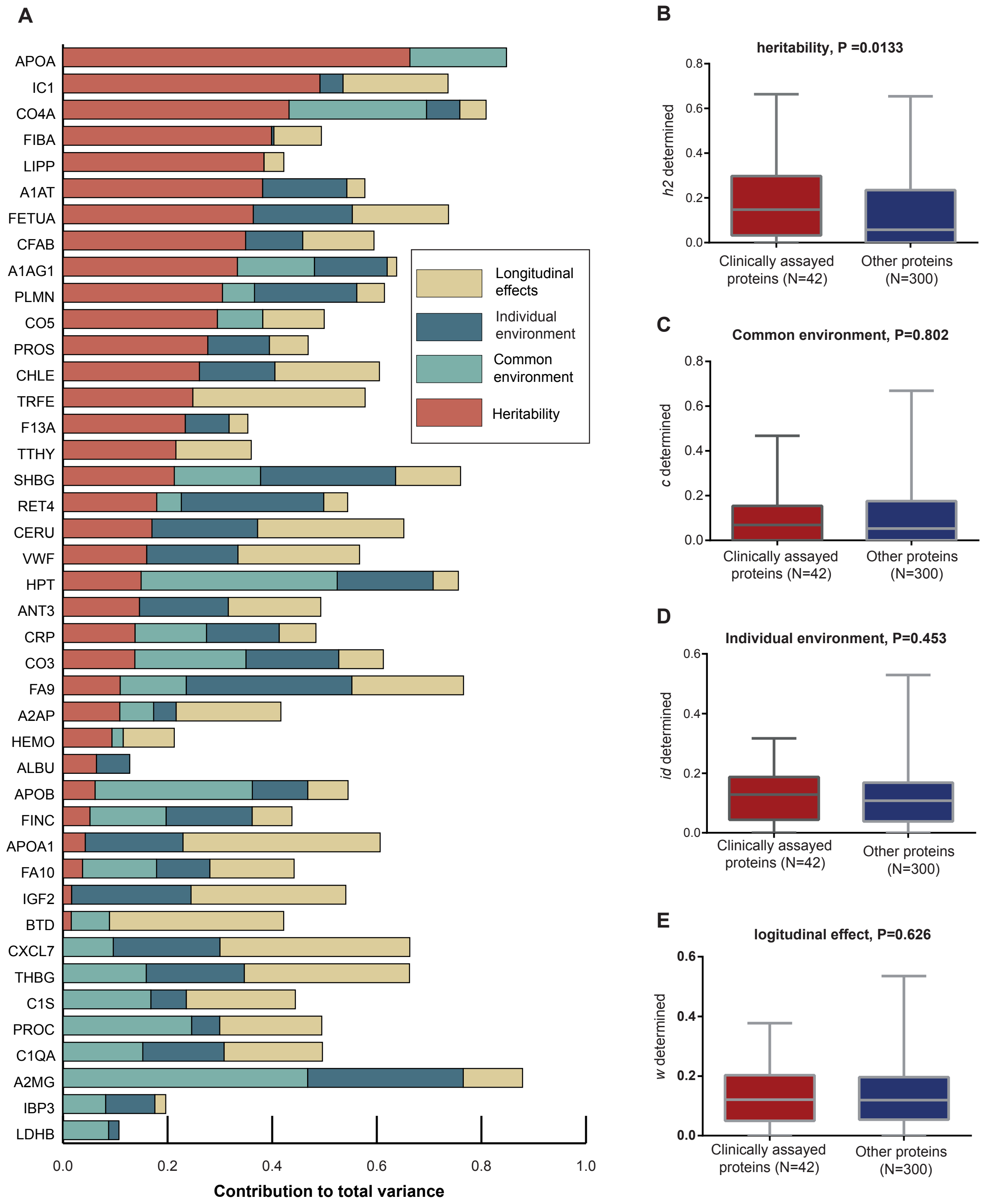

Figure S8. Variance analysis of detected clinically assayed proteins whose measurement were cleared or approved by FDA in human blood (in short, clinically assayed proteins).

(A) Variances decomposition results of 42 detected clinically assayed proteins according to FDA approval. **(B-E)** Comparison between clinically assayed proteins and other plasma proteins regarding the determined contribution percentage of heritability, common environment, individual environment and longitudinal aging effect in explaining protein phenotypic variances. We observed the overall lower variability for the levels of these clinically assayed proteins as illustrated in Fig. 4F, which could be partially ascribed to the higher heritability of these proteins.

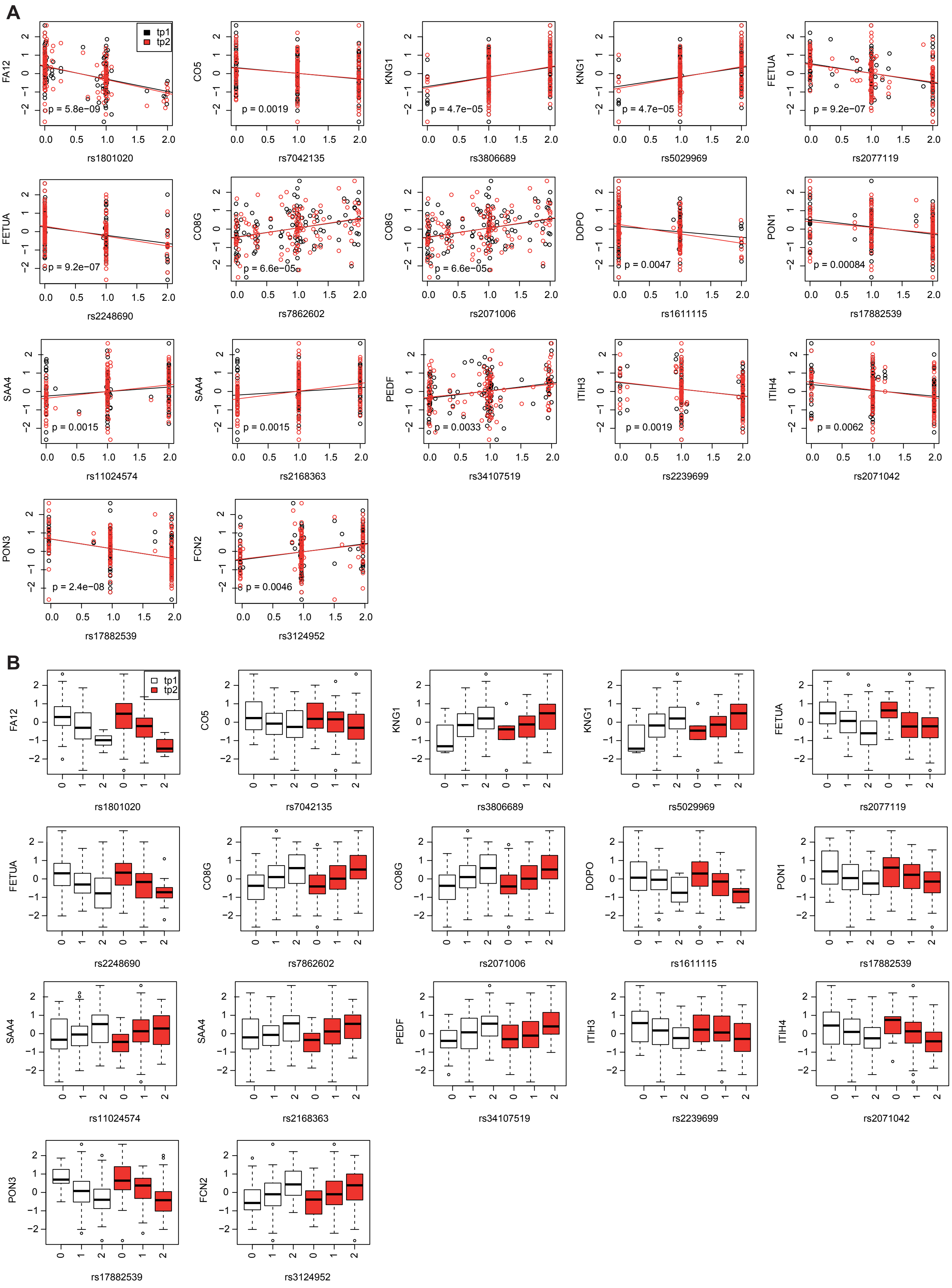
A
tp1
tp2
FA12
p = 5.8e−09
rs1801020
CO5
p = 0.0019
rs7042135
KNG1
p = 4.7e−05
rs3806689
KNG1
p = 4.7e−05
rs5029969
FETUA
p = 9.2e−07
rs2077119
FETUA
p = 9.2e−07
rs2248690
CO8G
p = 6.6e−05
rs7862602
CO8G
p = 6.6e−05
rs2071006
DOPO
p = 0.0047
rs1611115
PON1
p = 0.00084
rs17882539
SAA4
p = 0.0015
rs11024574
SAA4
p = 0.0015
rs2168363
PEDF
p = 0.0033
rs34107519
ITIH3
p = 0.0019
rs2239699
ITIH4
p = 0.0062
rs2071042
PON3
p = 2.4e−08
rs17882539
FCN2
p = 0.0046
rs3124952
B
tp1
tp2
FA12
rs1801020
CO5
rs7042135
KNG1
rs3806689
KNG1
rs5029969
FETUA
rs2077119
FETUA
rs2248690
CO8G
rs7862602
CO8G
rs2071006
DOPO
rs1611115
PON1
rs17882539
SAA4
rs11024574
SAA4
rs2168363
PEDF
rs34107519
ITIH3
rs2239699
ITIH4
rs2071042
PON3
rs17882539
FCN2
rs3124952

Figure S9. The pQTL linkages between plasma protein levels and genotyping data.

Note that we show the same information in both the uniform **(A)** dot plots and **(B)** boxplot. Consistent pQTL distributions were observed in both time pints (tp1 and tp2). In both cases, the x axis represents the genotype of the pQTL and the y axis the levels of a given protein.

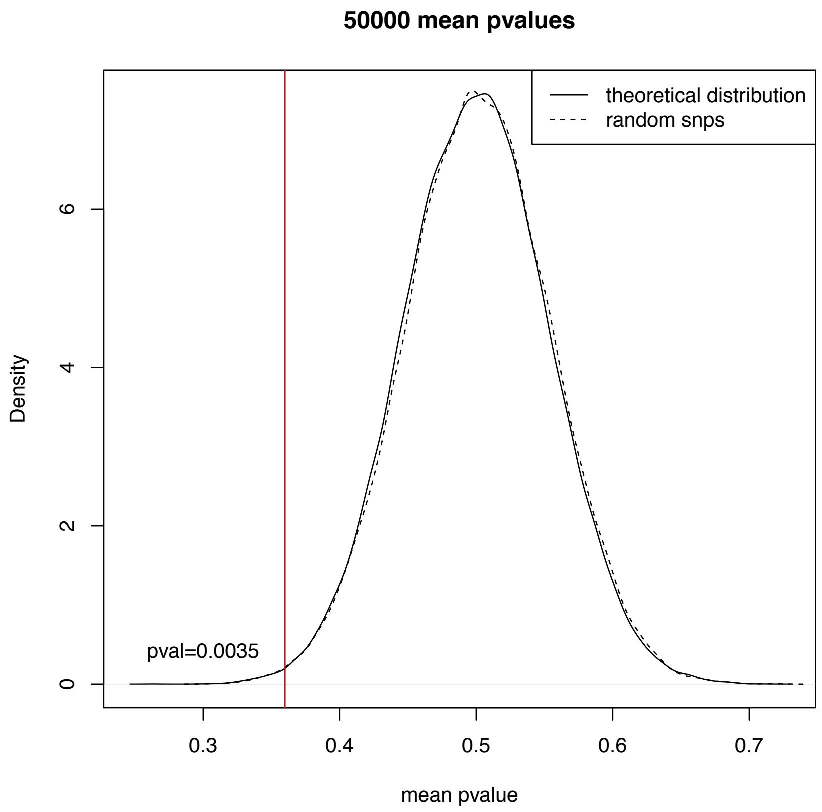

Figure S10. The analysis of statistical significance of pQTLs reported by Johannson's et al in our data set. Despite of the different sample cohorts, we estimated the mean of the pvalue of the Johanson's pQTL associations in our sample and compared this mean with the distribution of means expected if there were no signal in our sample. The solid black line and the dash line represent the distribution of 50000 random p values under the null hypothesis (no signal of Johannson's pQTLs existing in our sample*)* and the p values of the random SNPs. The red line denotes the averaged P values of Johannson's pQTLs in our sample.
