# Supplementary material for: Quantitative variability of 342 plasma proteins in a human twin population
Source: Mol Syst Biol. 2015 Feb 4;11(2):786. doi: 10.15252/msb.20145728 (PMC4358658; doi:10.15252/msb.20145728)
Supplement: Supplementary file 3 [file msb0011-0786-sd3.pdf]

molecular systems biology

Quantitative variability of 342 plasma proteins in a human twin population

Yansheng Liu, Alfonso Buil, Ben C Collins, Ludovic CJ Gillet, Lorenz C Blum, Lin-Yang Cheng, Olga Vitek, Jeppe Mouritsen, Genevieve Lachance, Tim D Spector, Emmanouil T. Dermitzakis, Ruedi Aebersold

Corresponding author: Ruedi Aebersold, ETH Zurich

Review timeline:

Submission date:	27 August 2014
Editorial Decision:	02 October 2014
Revision received:	23 November 2014
Editorial Decision:	23 December 2014
Revision received:	07 January 2015
Accepted:	12 January 2015

Editor: Thomas Lemberger

Transaction Report:

(Note: With the exception of the correction of typographical or spelling errors that could be a source of ambiguity, letters and reports are not edited. The original formatting of letters and referee reports may not be reflected in this compilation.)

1st Editorial Decision 02 October 2014

Thank you again for submitting your work to Molecular Systems Biology. We have now heard back from the three referees who agreed to evaluate your manuscript. As you will see from the reports below, the referees find the topic of your study of potential interest. They raise, however, a series of concerns on this work, which should be convincingly addressed in a revision of the study.

The reviewers are in general positive about the general approach of the study. The feel however that several of the conclusions do not seem to be sufficiently supported. Without repeating all the points listed by the reviewers, the major issues refer to the following:

- the conclusions on the impact of aging should be revised and considerably toned down unless corrections of potential confounding factors can be taken into account
- it seems that the statistical model and the interpretation of the effects included in the model should be revised according to the comments of reviewer #2
- potential biases due to a focus on high concentration range should be addressed
- an analysis of the contribution of PTM or protein structural variability to the observed concentration variability would be important.

© European Molecular Biology Organization

Reviewer #1:

This is a potentially interesting study in which the authors investigated quantitative variability in plasma proteins in a cohort consisting of 44 DZ and 72 MZ post-menopausal female twins with blood samples were drawn at two different time points starting at an average age of 57.8 years, and with a time interval between the two samplings of 5.2{plus minus} 1.4 years.
The authors conclude that the data indicates that inherent variability of protein levels varies significantly for different plasma proteins, and that the regulation of specific protein levels and biological processes are under tight genetic and/or aging control.

First a word of caution is needed about interpreting the findings as applicable to the entire plasma proteome given that the scope of the analysis is rather narrow compared to the vastness of the plasma proteome.

It would seem that the contribution of immunodepletion as a source of variability is not sufficiently well addressed. Also it is rather puzzling that the authors chose to limit their analysis to post-menopausal women with a span between the two time points representing only some 5 years. It is hard to appreciate how such a short span and to begin with concerning post-menopausal women would lead to significant changes in quantitative levels. One can only imagine then that a wider time span say encompassing menopause would lead to vast variations for which there is not good evidence. A comparison with a younger age group would have been informative in this regard even if it did not involve twins.

A major concern is that the study does not seem to account for structural variability in plasma protein as well as PTMs as potential sources of variability. Given the twin nature of the study these contributions to variability would be highly informative. Thus an integration of quantitative variability with genomic sequence information for the proteins investigated is likely to be very informative. We have known for decades about genetic/structural variability in plasma proteins and the significance of this study would be greatly enhanced by including an assessment of genetic variability in coding and non-coding sequences and their contribution to quantitative variability. Short of that the findings may be subject to conflicting interpretations.

Overall this study could be made more informative and more definitive by addressing the points raised in this review.

Reviewer #2:

Summary

The authors set out to estimate the heritability and other contributions of ~340 human plasma proteins using a longitudinal female twin study consisting of 72 MZ pairs / 44 DZ pairs. They identify protein QTL (pQTL) associated with genes coding for some of these proteins. They draw several conclusions regarding the relative contributions of genetics, shared environment, and aging.

The authors found that a large number of plasma proteins had substantial heritability. They identified 13 cis-SNPs associated with protein levels, and attempted to highlight the potential effect of aging on protein levels.

General remarks

Overall, the experimental design is sensible, and this twin study potentially provides a unique resource to assess the contribution of genetic and non-genetic factors to the variability of protein levels. Exploring the heritability of protein levels is important as this complements existing studies of other human molecular traits such as gene expression and metabolite levels. Importantly, due to technological advances in protein quantification, this study examined quite a large number of proteins, rather than be limited to a small number of proteins as in previous studies, such as Raffler et al 2013 (doi:10.1186/gm417). Such studies will be of interests to human genomicists and systems

© European Molecular Biology Organization

biologists.

However, there are substantial analytical issues for the study which are likely to affect all downstream analyses, especially those quantifying and interpreting any 'aging' effects. The limitations of the study should be very clearly discussed.

Major points

- The treatment of age in this study is a major issue which is likely to affect all analyses, and certainly those concerning aging effects. Here, there are two substantial issues.

The first is the model itself. The time is encoded as follow up, i.e., 0 at first visit and the time at second visit, which makes it hard to interpret whether there is an age effect. It would be far more interpretable to add the actual age as a fixed effect. In addition, in the linear mixed model, the ID "individual" matrix appears to be identical to the W "visit" matrix. For the shared environment matrix C, this essentially captures the fact that two individuals are twins, but it's hard to ascribe this to current shared environment (i.e., twins in their 60s are not likely to be living in the same house, for example), so the simple interpretation of this as "shared environment" needs to be a bit more nuanced. Also, it would make sense to make the "plate" effect a random effect rather than a fixed effect.

The second issue is that it is not possible to conclude from any time-varying effect that it is the result of aging and not other confounding. For example, it may be that older individuals tend to take more medication that in turn affect protein levels, or a myriad of other changes in diet, behavior or environment. This study does not attempt to control for any such potential confounders in the analysis, hence claiming that this is an age effect is premature.

A more realistic interpretation of the findings as well as an in-depth discussion of potential confounders and the limitations of the study is required, however, in the absence of appropriate control variables, this reviewer would prefer that all reference to aging effects as such be discarded.

- This study is rather small, a total of 131 individuals measured in two timepoints. It is likely that the variance components are estimated with substantial uncertainty which has not been reported in the study. Reporting the confidence interval for the estimates would allow assessment of the reliability of such estimates. Further, limiting the study to females based on the argument of excluding the gender variance seems heavy handed. A doubling of the sample size would be well worth adding another fixed effect "sex" to the model, in terms of power and reducing the uncertainty in the estimates.

- Figure 4C, 4D, 4E, what test are the p-values from? If it is a t-test, this would not be appropriate for comparing variances (heritabilities) as they are highly skewed and truncated at zero.

- The authors should use the Introduction to compare and contrast with Kato et al since it had a similar longitudinal proteome study design also using TwinsUK (Kato et al, 2011). Is there an overlap of TwinsUK samples between the two studies? If so, it would be useful to compare protein levels directly. Was the comparison between the current study and Kato et al using the same mixed model as the SWATH-MS? If not, this should be done as Kato et al utilized a different mixed model.

- The Discussion would be strengthened if it contrasted pQTLs with the Johannson study. Given the protein overlap, what pQTLs could have been detected? The Johannson study consisted of ~1,000 individuals, for those pQTLs not detected were there power issues? For example, were the strongest pQTL's in Johannson study, for A1AT (SERPINA1), technically detectable and if so was there appropriate power?

Minor points

- Within DZ twins, it would be informative to note whether there was a relationship between identity-by-descent and proteome similarity.

© European Molecular Biology Organization

- Introduction (pg2) "Genomic variation, modulated by lifestyle and environment, orchestrates the extensive phenotypic variability found in human populations." I think what the authors are trying to say is "The effects of genomic variation, modulated by lifestyle and environment, orchestrate the extensive phenotypic variability found in human populations."

- Introduction (pg2) "The quantification of heritability, i.e. the proportion of phenotypic variance attributable to additive genetic effects..." It should be clarified that the authors are referring to "narrow-sense heritability". 'Heritability' sometimes refers to broad-sense heritability, the full genetic component (inclusion of non-additive effects as well).

- Results (pg 7) "This discrepancy may be mainly ascribed to the much shorter temporal intervals of sampling used in their study (around 3 months), indicating that the natural aging process during the ~5 year period tested in the present study uncovered a profound impact of aging on plasma proteomic dynamics." This statement seems speculative in the absence of literature support, especially given very few time points (and potentially different samples). Further, if the major issues with regards to aging analyses were addressed, the authors could indeed test this in the data (phenotypic variance as a function of time interval between visits, though the time fixed effect will have to change).

- Figure 5A: The y-axis of the Manhattan plot should indicate that it is a P value. An FDR of 0.1 was used as cutoff, the corresponding p-value should be annotated on the Manhattan plot. How many SNPs were considered?

- Results (pg 11): A more thorough description of the '800 female twins' data is warranted.

- Discussion (pg 14): The paragraph beginning 'Understanding the underlying genetic determinants of biomarkers...' would benefit from less certain language. In particular, the simple logic that a genetic variant associated with a causal biomarker of a disease is itself necessarily a biomarker of the disease is false for the simple reason that there exist components of variance (e.g. the mixed model). Conversely, the discussion about how analysis of a non-causal biomarker like CRP would benefit from identification of pQTLs is unclear; it seems that the authors are trying to convey the concept of Mendelian randomisation. In terms of examples, it would be informative to also present cases where the authors' logic statements have not held.

- Methods (pg 20): What was the QC prior to imputation? Was there individual-level QC (missingness, outlier removal, etc)

- Methods (pg 21) "e is the residual effect" - it's not an "effect", it's just the residuals

- Methods (pg 22): It is not clear why the authors regress out 10 PCs of the protein levels if they have a linear mixed model already. This is apparently unnecessary as they can just take residuals or run a similar mixed model with protein levels as a function of the SNPs as a fixed effect.

- Results (pg 8): The pvalue notation, for example "P= 0.00032~2.60E-6", is odd and it is not clear it means.

- Please provide a better description of what is means to be a an "FDA cleared protein".

Spelling and grammar checks are needed. Some typos would include:
P21 "Tipe points", "familiar effect"
P11, "know genes"
P12 "familiar component"
P13 "committee" -> community?
P13 "know to be causal"
P20 "It is know that variance" ... "to non-normal"
P21 "familiar effect"

© European Molecular Biology Organization

Reviewer #3:

This is an elegant study concerning a highly multiplexed proteomic analysis of a unique cohort of plasma samples obtained from a monozygotic and dizygotic twin population taken at different times (~5 years apart). Such a cohort provides the powerful opportunity to begin to elucidate the significance of genetic vs aging vs diet/environment, etc. The analysis is the largest most comprehensive proteomic analysis to date for such a study. The investigators are thought leaders in the field and many of the methods pioneered by them. While the manuscript is very well written and will be of high interest to the readership, there are several opportunities missed that could have increased the impact of the paper.

Major Issues

1. I'm not sure the CAP oriented analysis is useful or if any of the data is meaningful and certainly the conclusions would need to be so circumspect that I don't think this adds to the paper: basically sampling 99 proteins out of thousands is of such low coverage (< 1%) that its impossible to conclude anything. Compounding this is that the abundance levels of the proteins chosen are fairly high abundance compared to many clinically used cancer markers/markers important in cancer biology today.

2. Following, the investigators really need to include a list of the concentrations of the proteins studied. An examination of the supplemental data make me concerned that most of the content is high abundance proteins with few in the microgram/ml range and very few in the ng/ml range. It is estimated that 90% of the plasma proteome exist in the sub fg/ml range. Thus there appears to be a huge percentage of the plasma proteome (especially in the low abundance range, which presumably contain the most interesting component) that was really unstudied here. IT would be very helpful to the reader to judge the data by having the average estimated concentration of the proteins enumerated in the supplemental table.

3. Following still- if the investigators could provide data that addresses if there is a concentration range bias in the study that could influence the final conclusions. For example, are those proteins that have the lowest concentration the most variable? most associated with environment changes? Same would go for the other categories. Some understanding of the percent of the proteome in each category that were in the mg/ml range, the microgram/ml range, nanogram/ml, femtogram/ml range etc. would be helpful.

A minor point: there are no FDA cleared proteins. there are FDA cleared biomarkers for diagnostic/prognostic purposes. As the reviewers of course know- the proteins exist in nature, not because the FDA approved them. Please consider renaming this cohort. Something like Protein biomarkers whose measurement has been approved by the FDA for clinical purposes.

1st Revision - authors' response 23 November 2014

Reviewer #1:

This is a potentially interesting study in which the authors investigated quantitative variability in plasma proteins in a cohort consisting of 44 DZ and 72 MZ post-menopausal female twins with blood samples were drawn at two different time points starting at an average age of 57.8 years, and with a time interval between the two samplings of 5.2(plus minus) 1.4 years.
The authors conclude that the data indicates that inherent variability of protein levels varies significantly for different plasma proteins, and that the regulation of specific protein levels and biological processes are under tight genetic and/or aging control.

First a word of caution is needed about interpreting the findings as applicable to the entire plasma proteome given that the scope of the analysis is rather narrow compared to the vastness of the plasma proteome.

<u>Author Reply:</u> *We agree with the reviewer that the 425 plasma proteins we detect on average per sample is far from the full plasma proteome. Nevertheless, the dataset is pioneering. For reference, credible mass spectrometric (MS) evidence exists for is ~2000 human plasma proteins. This evidence has been accumulated by combining 91 plasma datasets in PeptideAtlas database, some of which consist of literally hundreds of LC-MS/MS runs of the respective samples. In contrast to e.g. cell line derived data, even with the most advanced mass spectrometric methods it is challenging to identify a large number of plasma proteins due to the well-known technical challenges such as the high complexity and the extremely large dynamic range. Further, analysis of the twin data requires accurate quantification across multiple samples. Our data are the first mass spectrometric data at this scale that has the accuracy to detect smaller biological effects reliably. The generation of this unprecedented dataset was only achievable through the application of the new SWATH-MS technology which provides the reproducibility and tightness of quantification to support the twin study.*
To address the concern of the reviewer and to avoid the impression that we claim analysis of the full plasma proteome, in the revised manuscript, we first tuned down the respective expressions in the article and we also changed the title from "Quantitative variability of the plasma proteome in a human twin population" to "Quantitative variability of 342 plasma proteins in a human twin population".

It would seem that the contribution of immunodepletion as a source of variability is not sufficiently well addressed.

<u>Author Reply:</u> *We share the concern about variability potentially introduced by immune depletion of high abundance plasma proteins. Precisely for this reason we did NOT deplete the plasma samples for SWATH analyses. This is described in the method of "SWATH-MS measurement" (Please see Page 21 Para. 2 and at a small, separated paragraph of the Discussion (Page 17 Para. 3).*

We did use immunodepletion and SAX fractionation only for the shotgun experiments (i.e., the step for the generation of the spectral library). Here, all the matched ions in MS/MS spectrum are not affected by immunodepletion as they are confidently identified as peptides by the conventional shotgun proteomics.

We also add some further discussion related to the issue of depletion. We also mention the recent paper of Dayon et al. published at Journal of Proteome Research (Dayon et al, 2014) *where the authors assessed the result of the variability of depletion and buffer exchange on a robotic platform (See Page 17 Para. 3: "For future comparative studies, the relative variability derived from this study for certain plasma proteins (e.g., those interacting with Albumin) might need to be adjusted by factoring in the technical variations, e.g., those from immunodepletion (Dayon et al, 2014) or protein isolation steps, if indeed these steps are used")*

Also it is rather puzzling that the authors chose to limit their analysis to post-menopausal women with a span between the two time points representing only some 5 years. It is hard to appreciate how such a short span and to begin with concerning post-menopausal women would lead to significant changes in quantitative levels. One can only imagine then that a wider time span say encompassing menopause would lead to vast variations for which there is not good evidence. A comparison with a younger age group would have been informative in this regard even if it did not involve twins.

<u>Author Reply:</u> *Thanks for this comment. In the following we address the comment first by discussing the range of age and secondly the five-year age span investigated and then by improving the analysis of ageing/longitudinal sampling.*

1. The range of age investigated (~17% of the twins encompassed menopause during the two visits, as they are younger than others).

© European Molecular Biology Organization

- *For the concern that all the twins are post-menopausal, we would like to firstly note that, the age range of the female twins is actually rather broad. To make this point clearer we added the new Figure S1 of age distribution of all the twins in the study. Although 200 out of 232 the samples are from individuals older than 50 (ca. 50-78), there are 32 female twins (14%) are at the age of 38-49. Moreover, we now provide the detailed information we could extract from the twinUK database regarding menopausal status of all the 116 twins during the two visits (See "menopause status" in Table S1). We found that, (a) 66 out of 116 (i.e., 56.9%) of the twins are already at post-menopausal phase before visit 1 (average age =61.6 at visit 1); (b) 4 are at pre-menopausal phase at visit 1 and got menopause before visit 2 (average age of 47.3 at visit 1), and another 4 are going through menopause at visit 1 and were post menopause at visit 2 (average age of 49.5 at visit 1). Therefore 6.9% of the samples are known to encompass menopause. (c) Among the samples left where we do not have completed records of menopause status, 12 individuals are reported as "going through menopause at visit 1 and no data available at visit 2" (average age at 55.2 at visit 1). Considering the time interval of 5-6 years, these twins are also very likely to change the menopausal status between the two visits (12 out of 116, i.e., 10.3% of the twins). Therefore, at least 6.9-17.2% of the samples tested have the changed status of menopause, due to the broad range of age. We now mention this information in the result (Page 8 middle of Para. 3) and discussion (Page 17 line 5).*

- *Importantly, as we now point out in the discussion (Page 16 Para. 2), we selected this relatively older age range (from 30 to 78, while with 76% of the twins fall into the age between 50-70) for the clinical and medical consideration. Many systematic diseases such as cancer or diabetes begin to emerge at this age range. For example, over half (53%) of all cancers are diagnosed in adults aged 50-74 and over a third (36%) of all cancers are diagnosed in the elderly aged >75 in UK, 2009-2011 (Information from http://www.cancerresearchuk.org/cancer-info/cancerstats/incidence/age/). Though majority of these female twins are post-menopausal, or some of their plasma protein concentrations might be affected by menopause, they are anyway the best controls available as an "age-matched" group for the biomarker discovery studies of these diseases. In fact we suggest that, for studies aiming at discovering predictive or retrospective biomarkers, or to monitor the progress of the above-mentioned diseases, it is crucial to focus on this age range. Clearly, understanding the biological variability (as presented in this work) will be very beneficial for understanding the quantified plasma protein profiles in the biomarker studies. The degree of plasma protein variability so far has been essentially unknown.*

- *Of course, it would be ideal to have a longitudinal study with multiple sampling time point across the human life span as we also discussed. However, we are limited by the current capacity of sample throughput for the comprehensive large-scale proteomic analysis (considering the all the steps from reproducible sample preparation to mass spectrometry analysis and time consumed). In fact, to date our study is most comprehensive twin plasma proteomic study with the most individuals investigated based on our reproducible SWATH-MS technique. We discuss this also in Page 17 end of Para. 1.*

2. The five years age span investigated by longitudinal samples.

- *This reviewer worries that the 5-year span could be too short. In fact, the result suggests that the 5-year span is not too short, because a profound impact of aging on plasma proteomic dynamic levels was revealed. The results presented in Page 8 beginning of Para. 3 indicate that the longitudinal factor could explain 13.6% of the phenotype variance (that is 30.8%, or almost as high as one-third, of the biological stable variance), compared to 2.9% reported by Kato et al (Kato et al, 2011) where a time span of 3 months were used. Also, specific, clinically used protein analytes (Figure 4G), such as BTD or THBG, were found to be significantly altered throughout this five year longitudinal process. Their plasma levels were preferably affected by a longitudinal effect (>30%) compared to the heritability and environmental components.*

- *Most importantly, the judgment about whether the longitudinal span in a biomarker discovery study (which our study aims to support) is too short highly depends on the*

© European Molecular Biology Organization

disease targeted. For a slowly progressing disease such as prostate cancer, 5 years might be not enough to reveal the whole disease process. However, for many other general diseases, such as diabetes, Alzheimer's disease and many cancer types, a span of 5 years is clinically and medically relevant. For example, for ovarian cancer, the serum concentrations of CA125, HE4, and mesothelin were reported to provide evidence of cancer 3 years before clinical diagnosis(Anderson et al, 2010). Also for Diabetic nephropathy (a progressive kidney disease), the urinary levels of collagen fragments were demonstrated as prominent biomarkers 3–5 years before onset of macro albuminuria(Zurbig et al, 2012). Moreover, in clinics the "5 year survival rate" is a routine predictor for cancer patient's survival after surgery. Therefore, we suggest that a span of ~5-year is clinically relevant and a suitable "predictive window" for many diseases. We add the concise discussion in Page 16 end of Para. 3.

- *The issue of sample storage and other confounding factors in proteomic practice might hinder the analysis of a longer span (say, >5-10 years). The extreme long-term sample storage and the resultant inevitable freeze-thaw cycle(s) might be important factors that researchers need to consider for plasma proteomic projects. For example, possible protein degradation might need to be thoroughly tested. Very few studies have looked into this problem due to the practical difficulties of long-term sample storage. Mitchell et al. evaluated the impact of storage time and freeze-thaw cycles on plasma samples used in MS-based biomarker discovery and found that a 4-year difference in long-term storage is still reliable and had no effect on plasma protein levels, while the multiple freeze-thaw cycles has higher effect on proteome degradation(Mitchell et al, 2005). This is consistent with our observation. However, the longer-term stability of many molecules is still poorly understood and Gillio-Meina et al. expressed the caution of extreme long-term sample procurement in a recent review (Gillio-Meina et al, 2013). In addition to pre-analytical issues, there are also biological confounding factors in longer-term analysis (e.g., the number of medications taken along with ageing, as communicated with reviewer2). To address these issues we have added discussion text in Page 16 from the last line.*

3. The improved analysis of ageing/longitudinal sampling in the revised version.
We also choose to thoroughly analyze the effect of the absolute age of twin individuals in our current data set. Specifically, we added the numbers of actual age as a fixed effect in our variance model of two visits respectively. This analysis leads to the new Supplementary Figure S7, which demonstrates that the actual age (38-78) has a minimal effect in the variance dissection, accounting for 0-1% of the quantitative variability for most plasma proteins (Supplementary Figure S7-C, D). Moreover, the heritability of the proteins inferred with and without the actual age component are excellently correlated (R=0.99 for both visits, Figure S7-E, F). Therefore, the relative ageing process (or more accurately, the longitudinal process including all the temporally variable factors) seems to be much more important than the actual age (38-78) in explaining the total phenotypic and biological variance in plasma proteome. Please see corresponding results added in Page 12 middle of Para. 1. With this result, we believe our analyses of ageing/longitudinal changes are significantly improved in the revision.

A major concern is that the study does not seem to account for structural variability in plasma protein as well as PTMs as potential sources of variability. Given the twin nature of the study these contributions to variability would be highly informative. Thus an integration of quantitative variability with genomic sequence information for the proteins investigated is likely to be very informative. We have known for decades about genetic/structural variability in plasma proteins and the significance of this study would be greatly enhanced by including an assessment of genetic variability in coding and non-coding sequences and their contribution to quantitative variability. Short of that the findings may be subject to conflicting interpretations.

<u>*Author Reply:*</u> *We understand the reviewer's concern and agree that checking the variability regarding PTM and protein structures will provide useful information. Therefore in the revised paper, we carefully address this comment from technical and biological perspectives.*

1. PTMs and their variability: technical

In most standard proteomics workflows modifications are not routinely included in the database search. This is due to specific technical reasons. The first is the difficulty in controlling the false discovery rate (FDR) due to the massive expansion of the search space if PTM's are included. In particular, the inclusion of multiple PTM's that are searched combinatorially, expands the search space to a degree that makes the confident assignment of spectra to peptide sequences difficult. The second reason is the observation that modified peptides are in the minority in a complex sample and that they are generally best analyzed after enrichment from the primary digest. In our case, we even have further difficulties in directly analyzing PTMs in SWATH maps because we do not have the targeted MS assay for modified peptides. Therefore, to address the issue raised by the reviewer, we used an alternative approach to address the technical impact of peptide modifications in our variance analysis by a survey of reported modification sites in human Swiss-Prot database.

Specifically (See Page 7 Para. 2) we checked if the possible modifications of certain peptides could affect the variance decomposition results of the plasma proteins. We found only <5% (29 out of 595) of the peptides included in our model as protein quantitative evidence could be possibly modified according to Swiss-Prot database with any type modification site reported in human proteome (Supplementary Table S2 and Supplementary Figure S3A). These modifications include phosphorylation, acetylation, glycosylation, hydroxylation, methylation and others. Even for this minority of peptides (<5%), they achieved the identical results as other peptides in determining the contribution of family components to the total variance of the 12 proteins (R=0.83, P=0.00081, Supplementary Figure S3B). This analysis thus suggests that the peptides included in our model for protein quantification are dominated by their naked forms and the peptide-level modification status has an undetectable and negligible technical effect in our results.

Finally, we would like to communicate with the reviewer that algorithms for protein modification by SWATH-MS are being developed in the group, which may be applied to the same plasma data presented here in the future. However, these algorithmic and software development projects are not at a stage where they could be applied routinely.

2. PTMs, protein structures and their variability: biological.
We also mapped our protein list to Swiss-Prot for specific modifications and to Pfam database for the domain annotations. In the list of plasma proteins there are 170 proteins that can be potentially glycosylated, 148 proteins that can be phosphorylated, 89 proteins that can be acetylated and 155 proteins that could contain disulfide bond. The top five mostly identified Pfam domains are "Trypsin" (N=23 proteins), "V-set" (N=18), "Sushi" (N=18), "Serpin" (N=13) and "EGF" (N=11). Somewhat interestingly (See Supplementary Figure S6), we found that the proteins with different modification potential in the human proteome seem to have diverse regulation dependency on genetics or longitudinal factors. Those proteins annotated as "Glycoproteins" (Figure 4D) and with "disulfide bond" are more strongly regulated by genetics and less strongly affected by longitudinal factors, while the proteins with "Phosphoprotein" or "Acetylation" annotations show reverse trend of the regulation (Supplementary Figure S6 A-D, P<0.01 or P<0.05). The shared regulation trends are partially ascribed to the overlapping proteins between lists, i.e., "Glycoproteins" list is highly overlapping with "disulfide bond" proteins whereas the "Phosphoproteins" list is highly overlapping with the list of "acetylated" proteins (Supplementary Figure S6 E). These data are now added in the sections indicated above. Please also see Page 11 Para.2 for the relevant result.

For Pfam analyses (Supplementary Figure S6 F), the small number of each domain type annotated prohibited the significance calculation of regulation effectors. Therefore, we only plotted the average contributions of the four biological components for each of the five domain categories. The data suggest that the V-set domains are more strongly regulated by family component (heritability + shared environment, Supplementary Figure S6F) than other Pfam domains. Considering the fact that V-set domains are mainly found in diverse immunoglobulin light and heavy chains and in several T-cell receptors in human blood, we suggest that this trend might be related to the protein functions (rather than the domain itself), as the functional cluster of "immune response" are revealed to have a high degree of heritability (Figure 4A).

With the discussions above and the consideration that we did not directly measure the protein modification and structure in the current study, to address the issue raised by the reviewer we added

© European Molecular Biology Organization

a small, conservative paragraph for the biological perspective at Page 11 middle of Para 2. Specifically, the new text reads: "Furthermore, considering the proteins overlapping annotation classes and the fact that we did not directly measure any protein modification and structure in the current study, we suggest that further direct studies are crucial to conclude whether different protein modifications or structures indeed harbor diverse genetic or longitudinal regulation dependency."

3. Genetic variability on plasma protein concentration.
The association of genetic variability with plasma protein levels was already analyzed by our pQTL analysis (the association of SNPs at the genome with protein levels, see Supplementary Table S4 and Figure 5). We observed that most of the discovered pQTLs lie in regulatory regions (11 of the pQTL associations are at 5-upstream, 3 are at the intronic region) and only 2 were in the coding region, but synonymous. This result is also briefly mentioned in Page 13 end of Para. 2.

Overall this study could be made more informative and more definitive by addressing the points raised in this review.

Reviewer #2:

Summary

The authors set out to estimate the heritability and other contributions of ~340 human plasma proteins using a longitudinal female twin study consisting of 72 MZ pairs / 44 DZ pairs. They identify protein QTL (pQTL) associated with genes coding for some of these proteins. They draw several conclusions regarding the relative contributions of genetics, shared environment, and aging.

The authors found that a large number of plasma proteins had substantial heritability. They identified 13 cis-SNPs associated with protein levels, and attempted to highlight the potential effect of aging on protein levels.

General remarks

Overall, the experimental design is sensible, and this twin study potentially provides a unique resource to assess the contribution of genetic and non-genetic factors to the variability of protein levels. Exploring the heritability of protein levels is important as this complements existing studies of other human molecular traits such as gene expression and metabolite levels. Importantly, due to technological advances in protein quantification, this study examined quite a large number of proteins, rather than be limited to a small number of proteins as in previous studies, such as Raffler et al 2013 (doi:10.1186/gm417). Such studies will be of interests to human genomicists and systems biologists.

However, there are substantial analytical issues for the study which are likely to affect all downstream analyses, especially those quantifying and interpreting any 'aging' effects. The limitations of the study should be very clearly discussed.

Major points

- The treatment of age in this study is a major issue which is likely to affect all analyses, and certainly those concerning aging effects. Here, there are two substantial issues.

© European Molecular Biology Organization

The first is the model itself. The time is encoded as follow up, i.e., 0 at first visit and the time at second visit, which makes it hard to interpret whether there is an age effect. It would be far more interpretable to add the actual age as a fixed effect. In addition, in the linear mixed model, the ID "individual" matrix appears to be identical to the W "visit" matrix. For the shared environment matrix C, this essentially captures the fact that two individuals are twins, but it's hard to ascribe this to current shared environment (i.e., twins in their 60s are not likely to be living in the same house, for example), so the simple interpretation of this as "shared environment" needs to be a bit more nuanced. Also, it would make sense to make the "plate" effect a random effect rather than a fixed effect.

<u>Author Reply:</u> *We appreciate the comments from the reviewer and we explain better why we decided to use the model we used and the changes implemented to address the issues raised.*

1. In this study we are more interested in how the protein levels change in an individual as the individual ages than in the population change of the protein level with age. That is why we modeled the change in age per individual instead of the actual age of the individual. Since we recognize that the actual age could be important, we tested the effect of age on the protein levels for each time point separately (Supplementary Figure S7). We observed that for most proteins the effect of age is negligible and that the estimates of heritability are almost identical in the models with age and without age (correlation of 99%). We added this information to the manuscript. See results in Page 12 middle of Para. 1. Some discussion can be found in Page 17 Para.1 Line 9.

2. Regarding the ID and W matrices, we thank the reviewer for pointing out this mistake. ID and W are two different matrices. The ID matrix takes into account that the same individual is measured in TP1 and TP2. The W matrix takes into account that given a time point, the two sisters went together to take the samples. The calculations were done with the right matrix and the mistake was only in the matrix description in the text. We correct this mistake in the manuscript.

3. It is true that adult twins do not necessarily share environments. Still, the C matrix captures the fact that twins are raised together in a way that they share many more feature that non-twin siblings are expected to share (including the 9 months of gestation). It is also known that twins share more habits in the adult life than non-twin siblings. These similarities among twins can be confounded with genetic effects. The use of the C matrix joined with the IBD matrix helps to not overestimate heritability in twin studies. In the literature about twin studies it has been called shared environment for years. Therefore, we added a sentence to describe this component to avoid confusion (Page 7 Para. 1 Line 2).

4. We considered making the plate effect a random effect. However, the complexity of the model is very high (it already has 4 random effects) and the estimate of the random effects is difficult with our sample size. Moreover, there are only 3 plates, so we strongly believe that it is better to treat the plate effect as a factor with three levels (fixed effect).

The second issue is that it is not possible to conclude from any time-varying effect that it is the result of aging and not other confounding. For example, it may be that older individuals tend to take more medication that in turn affect protein levels, or a myriad of other changes in diet, behavior or environment. This study does not attempt to control for any such potential confounders in the analysis, hence claiming that this is an age effect is premature. A more realistic interpretation of the findings as well as an in-depth discussion of potential confounders and the limitations of the study is required, however, in the absence of appropriate control variables, this reviewer would prefer that all reference to aging effects as such be discarded.

<u>Author reply:</u> *We agree with the statement of the reviewer. Aging is a complex process that involves biological and sociological aspects. Since is not possible to take into account all the factors that change in time, we decided to follow the reviewer's advice and we changed the term aging for "longitudinal change" and we generally tone down the conclusions regarding impact of "aging" to the summary of "longitudinal or temporal effects".*

Moreover, we looked at the information that we could obtain from the samples to see if there were

distinctive changes between visit time points (TP1 and TP2) that could be considered confounding effects. We found several individuals suffering from diabetes type 2, but all of were already diabetic at TP1. We also found several individuals with cancer, but only in two cases did the cancer occur in the interval between TP1 and TP2. The situation regarding the intake of medicines it is more complicated and there is a higher degree of variability. It is difficult to draw a firm conclusion, but we cannot discard the possibility that intake of some medicines affected proteins levels between the two time points. We added this information as supplementary table S1 and presented it in the paper. See Page 8 from middle of Para. 3: "We also carefully checked the existence of other discernable longitudinal factors besides an ageing effect (Supplementary Table S1). We found that two individuals (i.e., 1.7% of 116 twins) in the cohort who developed cancer between the two visits; and at least 6.9-17.2% of the samples had changed menopausal status at the time of the two visits. A total of 15 (i.e., 12.9% of 116) twins had confirmed type II diabetes before visit one. No individual developed new diabetes type II at visit 2 in this cohort. According to the usage of four types of common medications (corticosteroids, thyroxine, statins and antihypertensives), we found that the twins tended to take more medications at the second visit (an average of 0.38 medications per person at visit 1 versus 0.53 medications per person at visit 2, P = 0.0125)." We also added the comment text on Page 8 Para 3 Line 5: "...indicating that the natural aging process together with other longitudinally unstable factors during the ~5 year period tested in the present study in total uncovered a profound impact of a relatively long-term, temporal changes on human plasma proteomic dynamics. "

- This study is rather small, a total of 131 individuals measured in two time points. It is likely that the variance components are estimated with substantial uncertainty which has not been reported in the study. Reporting the confidence interval for the estimates would allow assessment of the reliability of such estimates.

<u>Author reply:</u> *Given the sample size of our study, the estimates of heritability are not very precise. Following the reviewer's comment, we now added the confidence intervals to our heritability estimates (Supplementary Table S3).*

-Further, limiting the study to females based on the argument of excluding the gender variance seems heavy handed. A doubling of the sample size would be well worth adding another fixed effect "sex" to the model, in terms of power and reducing the uncertainty in the estimates.

<u>Author reply:</u> *As a general principle, we agree with the reviewer that it would be great to double the sample size and to include males to determine gender effects. However, availability of relevant samples is not always possible. Quantifying the plasma proteome in more than 200 samples is already a very significant effort that took several months to accomplish and is in fact, at this level of depth and reproducibility unprecedented in the proteomics literature. We like to highlight the value of the magnitude of this study in the proteomics field. Proteins predominantly determine the biochemical state of biological specimens and proteomic variation is therefore thought to be closely associated with phenotypic variation and adding a complementary component to the corresponding, nucleic acid-based indicators (Wu et al, 2013) and plasma protein levels cannot be inferred from transcript studies. Extending this comment, it would be ideal to have a longitudinal study with multiple sampling time point across the human life span as we also discussed. However, we are limited by the current capacity of sample throughput for the comprehensive large-scale proteomic analysis (considering the steps from reproducible sample preparation to mass spectrometry analysis and time consumed). In fact we would hope it can be appreciated that our study is the to date most comprehensive twin plasma proteomic study with the highest number of twin individuals investigated based on our sensitive, reproducible SWATH-MS technique and the unique, well-controlled experimental design. This is a significant achievement in proteomic field. Using women only in the study can limit the generalization of the conclusions for the general population but it still allows us to learn some biology. For example, the previous gene expression and methylation studies of twinUK with higher sample throughput (800-1000 twin individuals analyzed due to the high throughput of the genomic methods) also exclusively used the female individuals of the identical age range (Grundberg et al, 2012)(Buil et al, in press)(Glass et al, 2013) . We added some of the relevant discussions at Page 16 end of Para. 2. Finally, in a very recent study (Enroth et al, 2014) using ~1000 non-twin individuals, only four plasma proteins (among 92 tested) were identified as*

sex associated. This could also indicates that much more samples might be needed to extend our variability results to male twins to uncover plasma proteins associated with gender.

- Figure 4C, 4D, 4E, what test are the p-values from? If it is a t-test, this would not be appropriate for comparing variances (heritabilities) as they are highly skewed and truncated at zero.

Author reply: p-values in Figure 4 come from a t-test. Since we agree with the reviewer's comment, we recalculated the p-values using the non-parametric Wilcoxon rank sum test and report the new p-values. We clarify it in Methods (Page 27 Para. 3).

- The authors should use the Introduction to compare and contrast with Kato et al since it had a similar longitudinal proteome study design also using TwinsUK (Kato et al, 2011). Is there an overlap of TwinsUK samples between the two studies? If so, it would be useful to compare protein levels directly. Was the comparison between the current study and Kato et al using the same mixed model as the SWATH-MS? If not, this should be done as Kato et al utilized a different mixed model.

Author reply: Unfortunately, the samples in Kato et. al do not overlap with the samples used in our study, so we cannot make a protein-protein comparison. Regarding the model used in Kato et. al, we used the same conceptual model and the only difference is that Kato et. al estimated an individual visit effect using and extra random effect and the fact that they had two replicate aliquots per visit. Since we have a single aliquot per visit we do not need to estimate this effect. Other than that, the two models are equivalent. To make it clear, we now add this note at Page 8 Para. 3 Line 3.

- The Discussion would be strengthened if it contrasted pQTLs with the Johannson study. Given the protein overlap, what pQTLs could have been detected? The Johannson study consisted of ~1,000 individuals, for those pQTLs not detected were there power issues? For example, were the strongest pQTL's in Johannson study, for A1AT (SERPINA1), technically detectable and if so was there appropriate power?

Author reply: This is a very reasonable question. Johannson et al has a larger sample cohort (around 1000) and thus more power to detect pqtls as long as we do not consider the better reproducibility and quantitative accuracy provided by SWATH-MS.

To investigate if the fact that we do not find the pQTLs detected by Johannson is a matter of power we looked at the p-values of the association of Johanson's pQTLs in our study and looked if there is an enrichment for small p-values. The expectation is that if the Johannson pQTLs are not present in our sample, the distribution of p-values would be flat (a uniform 0,1). However, if the Johanson's pQTLs are present in our sample even though they may not be statistically significant, we should see an enrichment of small p-values in our sample. We estimated the mean of the p-value of the Johannson's pQTL associations in our sample and compared this mean with the distribution of means expected if there were no signal in our sample. Supplementary Figure S10 shows the expected distribution of means under the null hypothesis (no signal of Johannson's pQTLs in our sample) and the actual observed mean. From there we can calculate a p-value (0.0035) that supports the idea that, with a larger sample, we would find some of the pQTLs described by Johannson et al. Finally, the totally distinct sample cohort may be a factor for this discrepancy between pQTL studies. We added this information and the figure to the manuscript (Page 14, Para. 2).

Minor points

- Within DZ twins, it would be informative to note whether there was a relationship between identity-by-descent and proteome similarity.

Author reply: We thought about introducing a new random effect in the model accounting for IBD similarity so that we would have an estimate of the amount of variance explained by genetic variants

© European Molecular Biology Organization

in cis. However, we understand that the model as is already has many random effects and with the small sample size that we have that test would be completely underpowered.

- Introduction (pg2) "Genomic variation, modulated by lifestyle and environment, orchestrates the extensive phenotypic variability found in human populations." I think what the authors are trying to say is "The effects of genomic variation, modulated by lifestyle and environment, orchestrate the extensive phenotypic variability found in human populations."

Author reply: We appreciate the comment and changed the text accordingly.

- Introduction (pg2) "The quantification of heritability, i.e. the proportion of phenotypic variance attributable to additive genetic effects..." It should be clarified that the authors are referring to "narrow-sense heritability". 'Heritability' sometimes refers to broad-sense heritability, the full genetic component (inclusion of non-additive effects as well).

Author reply: We appreciate the comment and changed the text accordingly.

- Results (pg 7) "This discrepancy may be mainly ascribed to the much shorter temporal intervals of sampling used in their study (around 3 months), indicating that the natural aging process during the ~5 year period tested in the present study uncovered a profound impact of aging on plasma proteomic dynamics." This statement seems speculative in the absence of literature support, especially given very few time points (and potentially different samples). Further, if the major issues with regards to aging analyses were addressed, the authors could indeed test this in the data (phenotypic variance as a function of time interval between visits, though the time fixed effect will have to change).

Author reply: We agree with the reviewer that we cannot confirm that the differences observed between the two studies are due to the 'natural aging process'. As we discuss in the second point of reviewer 2, we are measuring 'changes in time' and not necessarily aging effects. So, it is expected that in 5 year will be more changes in the proteome of an individual than in only 3 months. However we cannot assure that these changes are only due to the natural aging process. We rewrote this sentence in the manuscript to make it more accurate (Page 8 Para 3 Line 5) and again, we mentioned it in discussion (Page 17 Para 1 Line 6).

- Figure 5A: The y-axis of the Manhattan plot should indicate that it is a P value. An FDR of 0.1 was used as cutoff, the corresponding p-value should be annotated on the Manhattan plot. How many SNPs were considered?

Author reply: We changed the y axis in the plot. We added to the plot caption cutoff pvalue = 6.166E-3. We added to the manuscript that the final number of tested SNPs was 758.

Results (pg 11): A more thorough description of the '800 female twins' data is warranted.

Author reply: Our paper describing these data and the eQTL analysis has been recently accepted and it is now in press (Buil et al, in press). We hope the citation will be available at the end of the editorial process of this paper. We add a brief statement about this study in the result (Page 14, Para. 1) and in Page 27 Para. 2 (Method section).

- Discussion (pg 14): The paragraph beginning 'Understanding the underlying genetic determinants of biomarkers...' would benefit from less certain language. In particular, the simple logic that a genetic variant associated with a causal biomarker of a disease is itself necessarily a biomarker of the disease is false for the simple reason that there exist components of variance (e.g. the mixed model). Conversely, the discussion about how analysis of a non-causal biomarker like CRP would benefit from identification of pQTLs is unclear; it seems that the authors are trying to convey the concept of Mendelian randomisation. In terms of examples, it would be informative to also present cases where the authors' logic statements have not held.

Author reply: We slightly disagree with the reviewer at this point. We believe that a genetic variant associated with a causal biomarker of a disease is itself necessarily a biomarker of the disease. It is true, however that the degree of the association between the genetic variant and the biomarker will determine the quality of the genetic variant as a marker and that, in general, the original biomarker will be a better biomarker than the genetic variant. Still, we think that the general principle holds and that the approach of finding genetic variants related to causal biomarkers opens the path to the discovery of genetic biomarkers for diseases. We revise the paragraph to point out this caveat and make it less certain. Regarding the discussion about non-causal biomarkers, we rewrote the sentence to make it clearer (Page 8 Para. 2).

Methods (pg 20): What was the QC prior to imputation? Was there individual-level QC (missingness, outlier removal, etc)

Author reply: We extended the explanation of the QC protocol for genotypes in Method section of the manuscript.

The new text is:

"Our samples are a subset of the TwinsUK data set. SNP Genotyping of the TwinsUK dataset (N~6,000) was done with a combination of Illumina HumanHap300, HumanHap610Q and 1M-Duo chips and was performed by The Wellcome Trust Sanger Institute and National Eye Institute via NIH/CIDR. Similar exclusion criteria were applied to each of the three datasets separately. Sample exclusion criteria were: (i) sample call rate <98%, (ii) heterozygosity across all SNPs ≥2 s.d. from the sample mean; (iii) evidence of non-European ancestry as assessed by PCA comparison with HapMap3 populations; (iv) observed pairwise IBD probabilities suggestive of sample identity errors; (v). We corrected misclassified monozygotic and dizygotic twins based on IBD probabilities. SNPs exclusion criteria were (i) Hardy-Weinberg p-value<10−6, assessed in a set of unrelated samples; (ii) MAF<1%, assessed in a set of unrelated samples; (iii) SNP call rate <97% (SNPs with MAF≥5%) or < 99% (for 1% ≤ MAF < 5%). Prior to merging, we performed pairwise comparisons among the three datasets and further excluded SNPs and samples to avoid spurious genotyping effects, indentified as follows: (i) concordance at duplicate samples <1%; (ii) concordance at duplicate SNPs <1%; (iii) visual inspection of QQ plots for logistic regression applied to all pairwise dataset comparisons; (iv) Hardy-Weinberg p-value<10−6, assessed in a set of unrelated samples; (v) observed pairwise IBD probabilities suggestive of sample identity errors. We then merged the three datasets, keeping individuals typed at the largest number of SNPs when an individual was typed at two different arrays. Samples were imputed into the 1000 Genomes Phase 1 reference panel (data freeze, 10/11/2010) (Abecasis et al, 2012) using IMPUTE2 (Howie et al, 2009) and filtered (MAF<0.01, IMPUTE info value < 0.8 and HWE (p<1e-5)). We ended with 3552380 SNPs in 113 of our 116 individuals."

- Methods (pg 21) "e is the residual effect" - it's not an "effect", it's just the residuals
Author reply: We appreciate the comment and changed the text accordingly.

Methods (pg 22): It is not clear why the authors regress out 10 PCs of the protein levels if they have a linear mixed model already. This is apparently unnecessary as they can just take residuals or run a similar mixed model with protein levels as a function of the SNPs as a fixed effect.

Author reply: The idea of regressing the first PC of a big experimental dataset as a way to remove large batch effects has been successfully using in big expression data studies (Lappalainen et al, 2013). It has been demonstrated that this correction improves the power to find QTLs (Stegle et al, 2012). We changed the text in the manuscript to explain this point better.

Results (pg 8): The pvalue notation, for example "P= 0.00032~2.60E-6", is odd and it is not clear it means.

© European Molecular Biology Organization

Author reply: We appreciate the comment and changed them to the ranges of p-values. We made it clear in the manuscript.

Please provide a better description of what is means to be a an "FDA cleared protein".

Author reply: We appreciate the comment. In fact reviewer#3 has a similar suggestion to change this term. According to the original, highly cited paper (Anderson, 2010) which we also cited upon usage, these proteins are the biomarker analytes the measurement of which has been approved by US Food and Drug Administration (FDA) for clinical purpose. We now use the new short name of "clinically assayed protein" instead of "FDA cleared proteins" to avoid confusion. We also refined the description at the first usage in Page 5 Line 5 from bottom of Para. 2.

Spelling and grammar checks are needed. Some typos would include:
P21 "Tipe points", "familiar effect"
P11, "know genes"
P12 "familiar component"
P13 "committee" -> community?
P13 "know to be causal"
P20 "It is know that variance" ... "to non-normal"
P21 "familiar effect"

Author reply: We appreciate the comment and corrected these typos. Thanks.

Reviewer #3:

This is an elegant study concerning a highly multiplexed proteomic analysis of a unique cohort of plasma samples obtained from a monozygotic and dizygotic twin population taken at different times (~5 years apart). Such a cohort provides the powerful opportunity to begin to elucidate the significance of genetic vs aging vs diet/environment, etc. The analysis is the largest most comprehensive proteomic analysis to date for such a study. The investigators are thought leaders in the field and many of the methods pioneered by them. While the manuscript is very well written and will be of high interest to the readership, there are several opportunities missed that could have increased the impact of the paper.

Major Issues

1. I'm not sure the CAP oriented analysis is useful or if any of the data is meaningful and certainly the conclusions would need to be so circumspect that I don't think this adds to the paper: basically sampling 99 proteins out of thousands is of such low coverage (< 1%) that its impossible to conclude anything. Compounding this is that the abundance levels of the proteins chosen are fairly high abundance compared to many clinically used cancer markers/markers important in cancer biology today.

Author Reply: We are sure that here is a typo of "<10%" (as we detected 99 CAPs among ~1000 proteins). But we think the reviewer's comment is very fair, because of the low coverage of CAP proteins and the fact that perhaps cancer biology is also difficult to be revealed by the "blood window" with limited analytical depth. Therefore we choose to delete the variance analysis of CAP proteins and only mention it as a list for uncovering SWATH-MS analytical depth.

© European Molecular Biology Organization

2. Following, the investigators really need to include a list of the concentrations of the proteins studied. An examination of the supplemental data make me concerned that most of the content is high abundance proteins with few in the microgram/ml range and very few in the ng/ml range. It is estimated that 90% of the plasma proteome exist in the sub fg/ml range. Thus there appears to be a huge percentage of the plasma proteome (especially in the low abundance range, which presumably contain the most interesting component) that was really unstudied here. IT would be very helpful to the reader to judge the data by having the average estimated concentration of the proteins enumerated in the supplemental table.

Author Reply: *We agree with this comment.*

Firstly, although our method promisingly detects on average 425 plasma proteins per sample there are many other proteins in plasma of which most lie in the lower abundance range. Today, there is Mass Spectrometry (MS) evidence of ~2000 human plasma proteins according to PeptideAtlas where the results of many and very large mass spectrometry studies are combined. Therefore, in the revised manuscript, we changed the title from "Quantitative variability of the plasma proteome in a human twin population" to "Quantitative variability of 342 plasma proteins in a human twin population".

Secondly, following reviewer's advice, we now provide the estimated concentrations of human plasma proteins reported from PeptideAtlas (Farrah et al, 2011) (Huttenhain et al, 2012) in the final column of Supplementary Table S3. The concentration distribution of proteins is plotted in Supplementary Figure 2A.

3. Following still- if the investigators could provide data that addresses if there is a concentration range bias in the study that could influence the final conclusions. For example, are those proteins that have the lowest concentration the most variable? most associated with environment changes? Same would go for the other categories. Some understanding of the percent of the proteome in each category that were in the mg/ml range, the microgram/ml range, nanogram/ml, femtogram/ml range etc. would be helpful.

Author Reply: *This is a very good suggestion. In the previous version of the paper we only looked at the possible concentration bias on heritability. Following the advice here, we now add the Supplementary Figure S5 and revised the Figure 4B. We find these analyses to be very informative and even provide more biological insights. Specifically, in Supplementary Figure S5, we plot the protein variability and the determined contribution from heritability, shared environment, individual environment and longitudinal factors to the total variance and the total biological variance. Figure S5A indicates that most of low abundant proteins are indeed more variable, as the reviewer and we expected; however, there are a few high abundant proteins which are also highly variable, which make the P value of the correlation insignificant. Even more interestingly, as illustrated in Figure 5SB-E, the heritability, shared environment and individual environment show no significant correlation with plasma concentrations, while the longitudinal factors clearly showed a difference, suggesting the concentration variability of more abundant proteins are generally less effected by longitudinal factors such as ageing. This is quite an interesting and novel finding. Please note that the relative contributions of the four biological components in total biological variance are inferred by removing the "unexplained fraction" (that means, the technical and experimental bias associated with specific proteins, e.g., concentration ranges, are expected to be removed). Additionally, Figure 4B is just the extraction of heritability and longitudinal contributions of the total biological variance.*

As we detected few proteins below the nanogram/mL range, we used a dashed, red line to indicate the two concentration ranges of microgram/mL and nanogram/mL according to the reviewer's suggestion (Figure 4B & Figure S5). From this we can also clearly see the trend of lower contribution of longitudinal effect on plasma proteome variability. We believe the P value of the Pearson correlation is more informative than the boxplot in this case, because we have the different concentration information for individual proteins.

The corresponding result description can be found in a new paragraph (Page 10 Para. 2).

© European Molecular Biology Organization

A minor point: there are no FDA cleared proteins. there are FDA cleared biomarkers for diagnostic/prognostic purposes. As the reviewers of course know- the proteins exist in nature, not because the FDA approved them. Please consider renaming this cohort. Something like Protein biomarkers whose measurement has been approved by the FDA for clinical purposes.

Author reply: *We appreciate the comment. We now changed this term by following the reviewer's advice when we mention it (Page 5 Line 5 from bottom of Para. 2). For the short term that are used in the Figures, we now change "FDA cleared proteins" to "clinically assayed proteins".*

References of the reviewer response

Anderson GL, McIntosh M, Wu L, Barnett M, Goodman G, Thorpe JD, Bergan L, Thornquist MD, Scholler N, Kim N, O'Briant K, Drescher C, Urban N (2010) Assessing lead time of selected ovarian cancer biomarkers: a nested case-control study. *Journal of the National Cancer Institute* 102: 26-38

Anderson NL (2010) The clinical plasma proteome: a survey of clinical assays for proteins in plasma and serum. *Clinical chemistry* 56: 177-185

Dayon L, Nunez Galindo A, Corthesy J, Cominetti O, Kussmann M (2014) Comprehensive and Scalable Highly Automated MS-Based Proteomic Workflow for Clinical Biomarker Discovery in Human Plasma. *Journal of proteome research*

Enroth S, Johansson Å, Enroth SB, Gyllensten U (2014) Strong effects of genetic and lifestyle factors on biomarker variation and use of personalized cutoffs. *Nat Commun* 5

Farrah T, Deutsch EW, Omenn GS, Campbell DS, Sun Z, Bletz JA, Mallick P, Katz JE, Malmstrom J, Ossola R, Watts JD, Lin B, Zhang H, Moritz RL, Aebersold R (2011) A high-confidence human plasma proteome reference set with estimated concentrations in PeptideAtlas. *Molecular & cellular proteomics : MCP* 10: M110 006353

Gillio-Meina C, Cepinskas G, Cecchini EL, Fraser DD (2013) Translational research in pediatrics II: blood collection, processing, shipping, and storage. *Pediatrics* 131: 754-766

Glass D, Vinuela A, Davies MN, Ramasamy A, Parts L, Knowles D, Brown AA, Hedman AK, Small KS, Buil A, Grundberg E, Nica AC, Di Meglio P, Nestle FO, Ryten M, consortium UKBE, Mu Tc, Durbin R, McCarthy MI, Deloukas P et al (2013) Gene expression changes with age in skin, adipose tissue, blood and brain. *Genome biology* 14: R75

Grundberg E, Small KS, Hedman AK, Nica AC, Buil A, Keildson S, Bell JT, Yang TP, Meduri E, Barrett A, Nisbett J, Sekowska M, Wilk A, Shin SY, Glass D, Travers M, Min JL, Ring S, Ho K, Thorleifsson G et al (2012) Mapping cis- and trans-regulatory effects across multiple tissues in twins. *Nature genetics* 44: 1084-1089

Huttenhain R, Soste M, Selevsek N, Rost H, Sethi A, Carapito C, Farrah T, Deutsch EW, Kusebauch U, Moritz RL, Nimeus-Malmstrom E, Rinner O, Aebersold R (2012) Reproducible quantification of cancer-associated proteins in body fluids using targeted proteomics. *Science translational medicine* 4: 142ra194

Kato BS, Nicholson G, Neiman M, Rantalainen M, Holmes CC, Barrett A, Uhlen M, Nilsson P, Spector TD, Schwenk JM (2011) Variance decomposition of protein profiles from antibody arrays using a longitudinal twin model. *Proteome science* 9: 73

Lappalainen T, Sammeth M, Friedlander MR, t Hoen PA, Monlong J, Rivas MA, Gonzalez-Porta M, Kurbatova N, Griebel T, Ferreira PG, Barann M, Wieland T, Greger L, van Iterson M, Almlof J, Ribeca P, Pulyakhina I, Esser D, Giger T, Tikhonov A et al (2013) Transcriptome and genome sequencing uncovers functional variation in humans. *Nature* 501: 506-511

© European Molecular Biology Organization

Mitchell BL, Yasui Y, Li CI, Fitzpatrick AL, Lampe PD (2005) Impact of freeze-thaw cycles and storage time on plasma samples used in mass spectrometry based biomarker discovery projects. *Cancer informatics* 1: 98-104

Stegle O, Parts L, Piipari M, Winn J, Durbin R (2012) Using probabilistic estimation of expression residuals (PEER) to obtain increased power and interpretability of gene expression analyses. *Nature protocols* 7: 500-507

Wu L, Candille SI, Choi Y, Xie D, Jiang L, Li-Pook-Than J, Tang H, Snyder M (2013) Variation and genetic control of protein abundance in humans. *Nature* 499: 79-82

Zurbig P, Jerums G, Hovind P, Macisaac RJ, Mischak H, Nielsen SE, Panagiotopoulos S, Persson F, Rossing P (2012) Urinary proteomics for early diagnosis in diabetic nephropathy. *Diabetes* 61: 3304-3313

2nd Editorial Decision 23 December 2014

Thank you again for submitting your work to Molecular Systems Biology. We have now heard back from the three referees who accepted to evaluate the revised study. As you will see, the referees are now supportive and we are pleased to inform you that your manuscript will be accepted for publication pending the following final amendments:

- The reviewers raise a few minor issues and makes suggestions that we would kindly ask you to address with suitable edits.

- Please remove the password protection on the data and update the text of the Data availability section accordingly.

- We would suggest to move/copy the short statement on the accessibility to the genotypic data through TREC under the "Data availability" section.

Reviewer #2:

The authors have largely addressed my concerns, however I have a few additional comments:

- While the authors have generally toned down conclusions around aging, there are places in the text which are not consistent. In particular:
(1) pg9's 2nd paragraph where there are still multiple references to 'aging' that should be modified, as the authors advise, to longitudinal changes/effects
(2) Figure 4E & 4G

- The 95% CIs for the heritability estimates need to be included in Table 1 as well as Table S3.

- On pg8, the authors should provide a more informative comparison by making clear to the reader that Kato et al, the only previous similar twin study, also utilized TwinsUK and also only female twins.

- "Johannson" is misspelled throughout pg 14, paragraph 2.

Signed:
Michael Inouye
Gad Abraham

© European Molecular Biology Organization

Reviewer #3:

The authors have provided a substantially improved paper and have addressed my concerns. I am especially interested in the finding that the longitudinal factors appear to show a difference, and the concentration variability of more abundant proteins seem to be less effected by longitudinal factors such as aging. The only minor comment is that there still seem to be vestiges of the "FDA cleared proteins" in the figures and figure legends. The authors should review these and please modify accordingly.

2nd Revision - authors' response 07 January 2015

Reviewer #2:

The authors have largely addressed my concerns, however I have a few additional comments:

- While the authors have generally toned down conclusions around aging, there are places in the text which are not consistent. In particular:
(1) pg9's 2nd paragraph where there are still multiple references to 'aging' that should be modified, as the authors advise, to longitudinal changes/effects
(2) Figure 4E & 4G

Author reply: *Thanks. We have now changed all the terms of "aging" to "longitudinal changes/effects" in the corresponding places of (1) and (2) (Please see Page 9, Para.2; Page 12, 6th Line of Para.1; Page 13, end of Para.1; and Figure legends of Figure 4E and 4G).*

- The 95% CIs for the heritability estimates need to be included in Table 1 as well as Table S3.

Author reply: *We now include the 95% CIs for the heritability estimates also in Table 1 for those heritable proteins.*

- On pg8, the authors should provide a more informative comparison by making clear to the reader that Kato et al, the only previous similar twin study, also utilized TwinsUK and also only female twins.

Author reply: *We now add this information. Please see Page 8, Para 2: "…in which 58 plasma proteins were analyzed by using also a female twin cohort from Twins UK but an antibody bead-based technology (Kato et al, 2011)."*

- "Johannson" is misspelled throughout pg 14, paragraph 2.

Author reply: *We now corrected three times of misspelling of "Johannson" (to "Johansson"). Thanks!*

Reviewer #3:

The authors have provided a substantially improved paper and have addressed my concerns. I am especially interested in the finding that the longitudinal factors appear to show a difference, and the concentration variability of more abundant proteins seem to be less effected by longitudinal factors such as aging. The only minor comment is that there still seem to be vestiges of the "FDA cleared proteins" in the figures and figure legends. The authors should review these and please modify accordingly.

© European Molecular Biology Organization

<u>*Author reply*</u>*: Thanks. We now changed "FDA cleared proteins" in our Figure 4F (which we missed in the last revision) to "Clinically assayed proteins" and double-checked this terminology, so that it is the consistent in the legends, text and Figure S8.*

© European Molecular Biology Organization
